# Diagnostic and therapeutic optical imaging in cardiovascular diseases

**DOI:** 10.1016/j.isci.2024.111216

**Published:** 2024-10-22

**Authors:** Weiran Pang, Chuqi Yuan, Tianting Zhong, Xiazi Huang, Yue Pan, Junle Qu, Liming Nie, Yingying Zhou, Puxiang Lai

**Affiliations:** 1 Department of Biomedical Engineering, The Hong Kong Polytechnic University, Hong Kong SAR, China; 2 Medical Research Institute, Guangdong Provincial People’s Hospital (Guangdong Academy of Medical Sciences), Southern Medical University, Guangzhou 510080, China; 3 Guangdong Provincial Key Laboratory of Malignant Tumor Epigenetics and Gene Regulation, Medical Research Center, Sun Yat-Sen Memorial Hospital, Sun Yat-Sen University, Guangzhou 510120, China; 4 Key Laboratory of Optoelectronic Devices and Systems of Ministry of Education and Guangdong Province, College of Physics and Optoelectronic Engineering, Shenzhen 518060, China; 5 College of Professional and Continuing Education, The Hong Kong Polytechnic University, Hong Kong SAR, China; 6 The Joint Research Centre for Biosensing and Precision Theranostics, The Hong Kong Polytechnic University, Hong Kong SAR, China; 7 Nanchang Research Institute, Sun Yat-Sen University, Nanchang 330096, China

**Keywords:** Health sciences, Natural sciences, Optics

## Abstract

Cardiovascular disease (CVD) is one of the most prevalent health threats globally. Traditional diagnostic methods for CVDs, including electrocardiography, ultrasound, and cardiac magnetic resonance imaging, have inherent limitations in real-time monitoring and high-resolution visualization of cardiovascular pathophysiology. In recent years, optical imaging technology has gained considerable attention as a non-invasive, high-resolution, real-time monitoring solution in the study and diagnosis of CVD. This review discusses the latest advancements, and applications of optical techniques in cardiac imaging. We compare the advantages of optical imaging over traditional modalities and especially scrutinize techniques such as optical coherence tomography, photoacoustic imaging, and fluorescence imaging. We summarize their investigations in atherosclerosis, myocardial infarction, and heart valve disease, etc. Additionally, we discuss challenges like deep-tissue imaging and high spatiotemporal resolution adjustment, and review existing solutions such as multimodal integration, artificial intelligence, and enhanced optical probes. This article aims to drive further development in optical imaging technologies to provide more precise and efficient tools for early diagnosis, pathological mechanism exploration, and treatment of CVD.

## Graphical abstract

**Figure:**
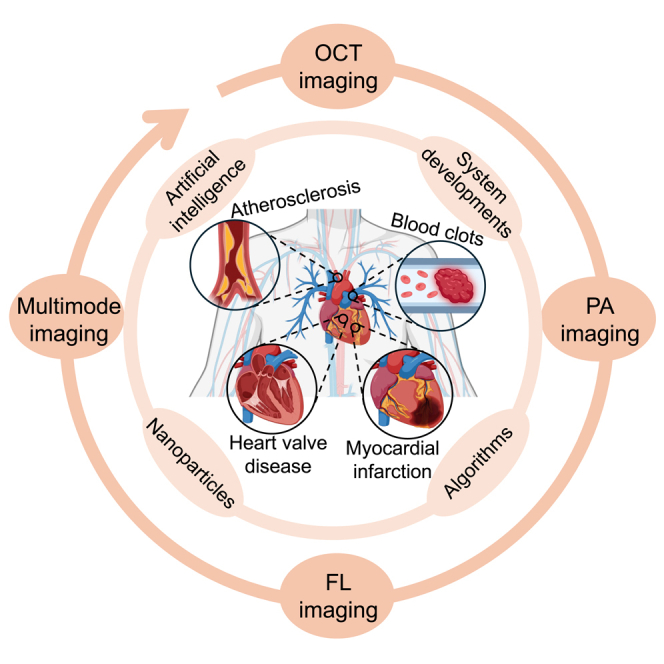

## Introduction

Cardiovascular disease (CVD) is the leading cause of mortality worldwide, accounting for over 31% of all fatalities. Conditions such as coronary artery disease (CAD), myocardial infarction (MI), heart failure, and arrhythmias pose substantial challenges in diagnosis, treatment, and management. Traditional modalities, including electrocardiography (ECG), echocardiography, digital subtraction angiography (DSA), and cardiac magnetic resonance imaging (CMR), have played crucial roles in assessing cardiac structure and function in clinic.^1^^,^^2^ Among them, ECG is the most common examination and the most affordable and simple way to diagnose heart disease. It is often used to check for myocardial ischemia, heart attacks, and arrhythmias. That said, it can only detect cardiomyopathy, but not whether there is damage to the heart’s structure or a defect in heart function. Echocardiography can clearly show the structure of the heart, such as the thickness of the heart walls, the size of the heart chambers, and the opening and closing of the heart valves. It can also show the movement of the heart wall and can more accurately determine the patient’s heart function. However, the resolution is relatively low in echocardiography. DSA is a means of vascular angiography and is the gold standard for the diagnosis of cardiovascular stenosis. It can diagnose the degree of blockage in the coronary arteries and help to unclog the vessels through interventional procedures when necessary. However, there are certain hazards involved with its use due to its invasiveness. CMR is a magnetic field scan to assess the location and size of the heart, and to understand the structure of the pericardium, coronary artery through angiography. However, it takes longer and costs more for examination than other approaches. Therefore, there is a growing need for more advanced imaging techniques that can provide detailed information about the underlying pathophysiology and guide targeted interventions.

In recent years, optical imaging has emerged as a promising field for studying CVDs. Various optical imaging modalities,^3^ such as optical coherence tomography (OCT),^4^ near-infrared spectroscopy (NIRS), and fluorescence imaging (FLI),^5^ as well as photoacoustic imaging (PAI),^6^^,^^7^ have been developed and applied to investigate cardiac morphology, blood flow dynamics, tissue oxygenation, and molecular processes. These techniques offer unique advantages, including high spatial resolution, real-time imaging capabilities, and the potential for molecular-level characterization (Table 1). OCT, for instance, enables high-resolution cross-sectional imaging of coronary arteries, allowing the identification of atherosclerotic plaques and assessment of their vulnerability. It has also been used to guide percutaneous coronary interventions (PCIs) and evaluate stent implantation outcomes. NIRS provides information about the lipid content in coronary plaques, aiding in the assessment of their stability. FLI techniques allow for the visualization of specific molecular targets involved in cardiac pathologies, such as inflammation, fibrosis, and apoptosis. PAI, built upon detection of laser-induced ultrasound, has shown benefits in imaging myocardial oxygenation, blood perfusion, and molecular signatures, and offers potentials for noninvasive characterization of ischemic heart disease and monitoring therapeutic interventions. Advancements in these light-based techniques have facilitated the improvement of functional imaging for assessing cardiac physiology, and the integration of these modalities has enabled multiparametric information for comprehensive CVDs diagnosis.

**Table 1:** Comparison of optical imaging modalities and traditional methods in cardiovascular applications

Imaging Modality	Spatial Resolution	Imaging Depth	Advantages	Disadvantages	Representative Works
ECG	N/A	N/A	• Non-invasive • Low cost • Widely available	• Limited structural information • No direct visualization of the heart	• Hedén et al. (1997)^176^: Standard 12-lead ECG for detection of myocardial ischemia, infarction, and arrhythmias.
Echocardiography	0.5–1 mm	10-15 cm	• Non-invasive • Real-time imaging • Assessment of cardiac structure and function	• Limited spatial resolution • Operator-dependent • Limited field of view	• Mitchell et al. (2019)^177^: Transthoracic echocardiography (TTE) for assessment of cardiac chambers, valves, and wall motion. • Peterson et al. (2003)^178^: Transesophageal echocardiography (TEE) for detailed visualization of valves and atria.
CMR	1-2 mm	Whole body	• High soft-tissue contrast • Non-invasive • Assessment of cardiac structure, function, and viability	• Long acquisition times • High cost • Contraindicated in patients with metallic implants	• Schelbert et al. (2010)^179^: Late gadolinium enhancement (LGE) for assessment of myocardial infarction and fibrosis.
DSA	0.1–0.2 mm	Whole body	• High spatial resolution • Gold standard for coronary artery visualization	• Invasive • Radiation exposure • Risk of complications	• Williams et al. (2016)^180^: Coronary angiography for diagnosis of coronary artery disease and guidance of interventions.
OCT	10-20 μm	1-2 mm	• High spatial resolution • Real-time imaging • Near histological level visualization	• Limited imaging depth • Requires catheterization for intravascular imaging	• Kang et al. (2023)^37^: OCT-guided PCI was noninferior to IVUS-guided PCI in target vessel-related outcomes at one year. • Lye et al. (2019)^43^: OCT imaging of human pulmonary vein-atrial junction, visualizing venous endothelium, myocardial sleeves, and fibrosis.
PAI	5-200 μm	1 mm-5 cm	• Deep tissue penetration • High spatial resolution • Molecular imaging capabilities • Multiparametric information	• Limited by optical attenuation and ultrasound reception • Quantitative analysis challenges	• Lin et al. (2018)^76^: Hemispherical PAI system for real-time volumetric imaging of whole isolated mouse hearts. • Ahn et al. (2023)^121^: Non-invasive monitoring of transient arterial vasoconstriction in acute hyperglycemia. • Ma et al. (2021)^101^: Ratiometric semiconducting polymer nanoparticles for PA imaging of O_2_ level within atherosclerotic lesions in pneumonia-complicated mice.
FLI	1 μm-1mm	<1 mm or whole body	• High sensitivity and specificity • Molecular targeting capabilities • Real-time imaging of biological processes	• Resolved images cannot have both depth and resolution simultaneously • Requires exogenous contrast agents	• Stein-Merlob et al. (2017)^137^: Detection of nanoparticle deposition in atheroma by NIR FLI. • Ortgies et al. (2019)^142^: Ag_2_S-ATII nanodots for NIR-II imaging of ischemic myocardium at different stages post-infarct. • Baugh et al. (2017)^145^: Providing a quantitative and sensitive readout of calcified nodule formation, especially in the context of CAVD.

This review is aimed to provide an overview of the recent advancements, applications, and future prospects of optical imaging in cardiovascular and cardiac visualization. We first introduce several commonly used optical imaging techniques for cardiac disease monitoring, including OCT, FLI, PAI, and afterglow imaging, and discuss their underlying principles, system configurations, and data processing. We then focus on the applications of these methods in multiple CVDs, pathophysiological research, and treatment monitoring, specifically examining studies and clinical applications related to atherosclerosis, MI, and valvular heart disease. Additionally, we address challenges associated with current optical imaging technologies, such as penetration into deep tissue, optical information processing, and contrast agent selection. Finally, we propose potential solutions and future perspectives of advanced cardiac imaging. Through this comprehensive review, it is aimed to promote further advancements in optical imaging technology in the field, providing more accurate and efficient tools for minimal-invasive or non-invasive diagnosis and treatment of CVDs.

## Optical coherence tomography in cardiovascular diseases

OCT, first developed around 1990,^8^ is a noninvasive imaging technique that utilizes optical coherence domain reflectometry to produce cross-sectional, tomographic images of biological tissues at micrometer-scale resolution. The principle of OCT is based on low coherence interferometry (LCI), which is same with Michelson interferometer.^8^ A typical OCT system consists of the following main components: light source, interferometer, probe, detector, signal processing and image reconstruction. Light from the source is split into two beams by a beam splitter, with one beam illuminating a reference mirror and the other illuminating the sample. Due to the low coherence of the light source, interference signals are produced only when the path difference between the sample reflected light and the reference mirror reflected light falls within the coherence length range. By scanning the reference mirror to change the path difference between the two beams, depth-resolved reflectance signals from different depths within the sample can be obtained, which can be used to reconstruct cross-sectional images of the sample’s structure.^9^

Currently, OCT technology is constantly evolving and has been successfully used in areas such as clinical ophthalmology. It has since proved to be effective in various fields, especially in cardiology,^10^ due to its high resolution and non-invasive nature. In the following sections, we will explore how OCT can be applied in diagnosing, treating, and researching cardiovascular conditions.

### OCT in coronary artery disease

Coronary artery disease (CAD) is a significant cardiovascular disorder characterized by the narrowing or blockage of coronary arteries, primarily due to the buildup of atherosclerotic plaques.^11^ As one of the diseases with high global morbidity and mortality, CAD faces substantial challenges in terms of early diagnosis, risk stratification, and treatment optimization.^12^ Traditional imaging techniques, such as angiography and intravascular ultrasound (IVUS), are limited in providing detailed information about the microstructure and composition of coronary plaques, which are essential for determining plaque vulnerability and guiding therapeutic interventions.^4^

OCT, with its high spatial resolution (10–20 μm) and ability to penetrate superficial arterial wall layers, has emerged as a promising technique for comprehensive CAD assessment.^13^^,^^14^^,^^15^ It enables *in vivo* near-histological visualization of coronary plaques, allowing accurate characterization of plaque morphology, composition, and vulnerability.^16^ OCT aids in the identification of hemodynamically significant stenoses and vulnerable plaques prone to rupture.^17^^,^^18^^,^^19^^,^^20^ These insights contribute to risk stratification and personalized management of CAD patients across a wide range of clinical scenarios, from diagnostic evaluation to treatment planning and optimization.^21^^,^^22^

#### Detection and characterization of coronary atherosclerotic plaques

**Figure 1:**
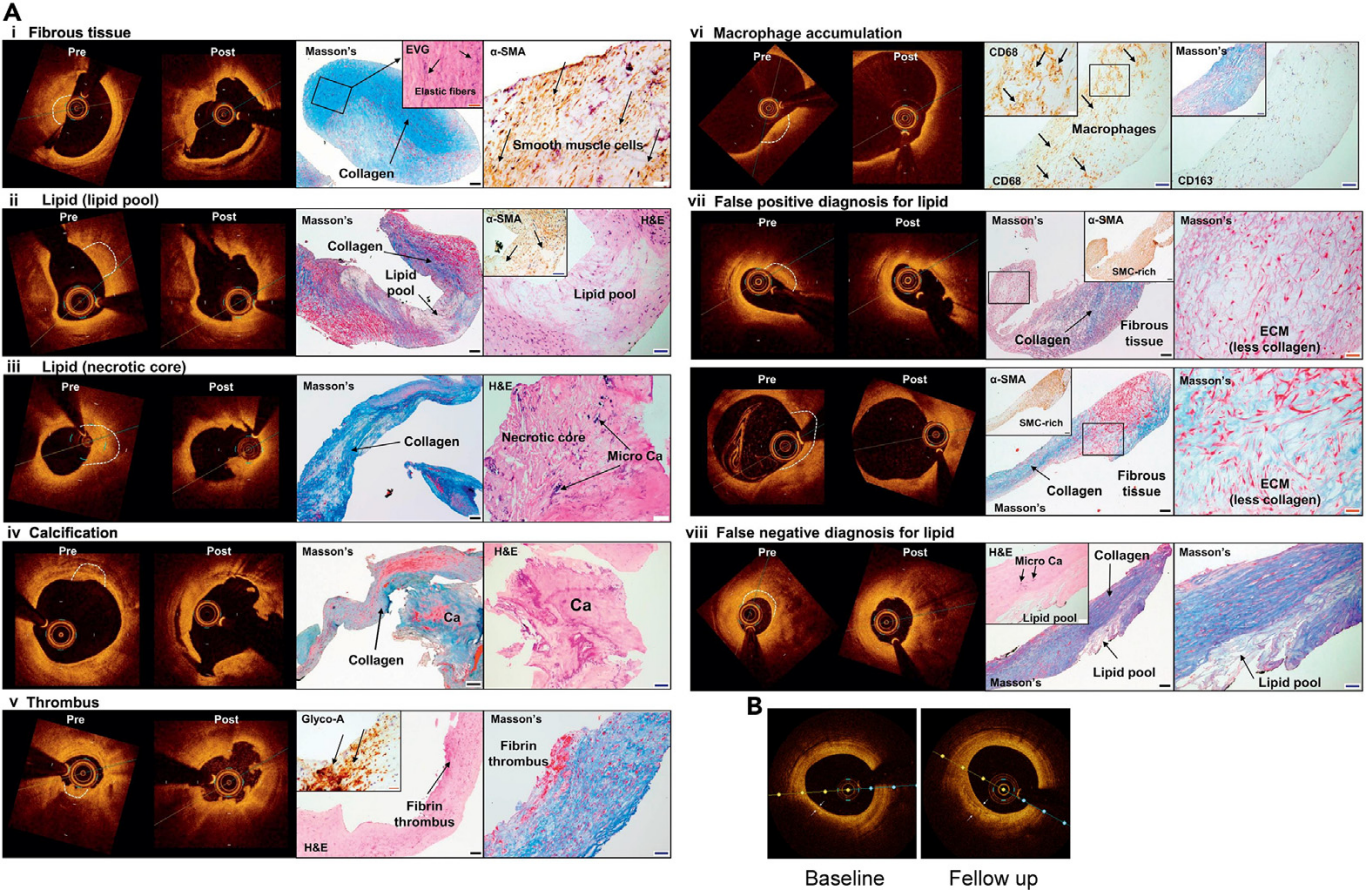
Detection and characterization of coronary atherosclerotic plaques with OCT (A) Representative and misinterpreted OCT images of excised tissues by DCA and corresponding histologic images showing various morphological characteristics. Reproduced with permission from.^28^ Copyright 2018 Elsevier. (B) Representative OCT images show changes in fibrous cap thickness between the baseline and follow-up in a patient with alirocumab. Reproduced with permission from.^29^ Copyright 2021 Springer Nature.

The capability of OCT in identifying histological features of atherosclerotic plaques *ex vivo* was first reported in 2002, which established a foundation for plaque characterization with OCT.^23^ Further research on autopsy specimens and *ex vivo* experiments confirmed that OCT could recognize the compositional and vulnerability characteristics of plaques.^24^^,^^25^^,^^26^ In 2010, the first *in vivo* study on plaque characterization using OCT was conducted, confirming its value in identifying fibrous cap thickness and plaque rupture, which are indicative of vulnerability.^27^ Then, in 2018, a pioneering *in vivo* study was reported to validate the diagnostic accuracy of OCT in characterizing human coronary atherosclerotic plaques by comparing OCT images with histopathology.^28^ The study enrolled 25 patients with stable angina pectoris who underwent directional coronary atherectomy (DCA). OCT imaging was performed before and after DCA, and the excised plaque tissues were histologically analyzed. The results demonstrated that OCT reliably predicts the presence of fibrous tissue, lipids, calcifications, and macrophage aggregations (Figure 1A). The sensitivity, specificity, positive predictive value, negative predictive value and predictive accuracy of OCT for lipids were 88.9%, 75.0%, 66.7%, 92.3%, and 80.0%; for calcifications were 50.0%, 100%, 100%, 91.3%, and 92.0%; and for macrophage aggregates were 85.7%, 88.9%, 75.0%, 94.1%, and 88.0%, respectively. However, it also showed a tendency for the over-detection of lipids, under-detection of calcifications and under-estimation of the deeper endothelial stroma, leading to potential misjudgments. Nevertheless, this study provides important *in vivo* evidence for the strengths and limitations of OCT in the tissue characterization of human coronary plaques, which has significant implications for the clinical application of OCT in the diagnosis and treatment of CAD. OCT shows promise in detecting high-risk vulnerable plaques. Researchers found that lesions in the fractional flow reserve (FFR)-positive group had smaller minimum lumen areas and a higher proportion of lipid plaques, suggesting that OCT and FFR can be used complementarily to identify high-risk lesions.^30^ Moreover, in a comprehensive meta-analysis conducted by Ramasamy et al., the diagnostic performance of OCT and IVUS in assessing FFR-related stenosis was compared. The results demonstrated that OCT-derived minimum lumen area (MLA) exhibited superior diagnostic accuracy compared to IVUS-MLA, with a significantly higher specificity and a greater area under the ROC curve in detecting FFR<0.80 stenosis. Subgroup analysis revealed that OCT-MLA maintained its diagnostic advantage over IVUS-MLA regardless of vessel size. These findings provide strong evidence supporting the preferential use of OCT for functional assessment of coronary stenosis in clinical practice, highlighting its superior specificity and accuracy in identifying hemodynamically significant lesions.^31^

**Figure 2:**
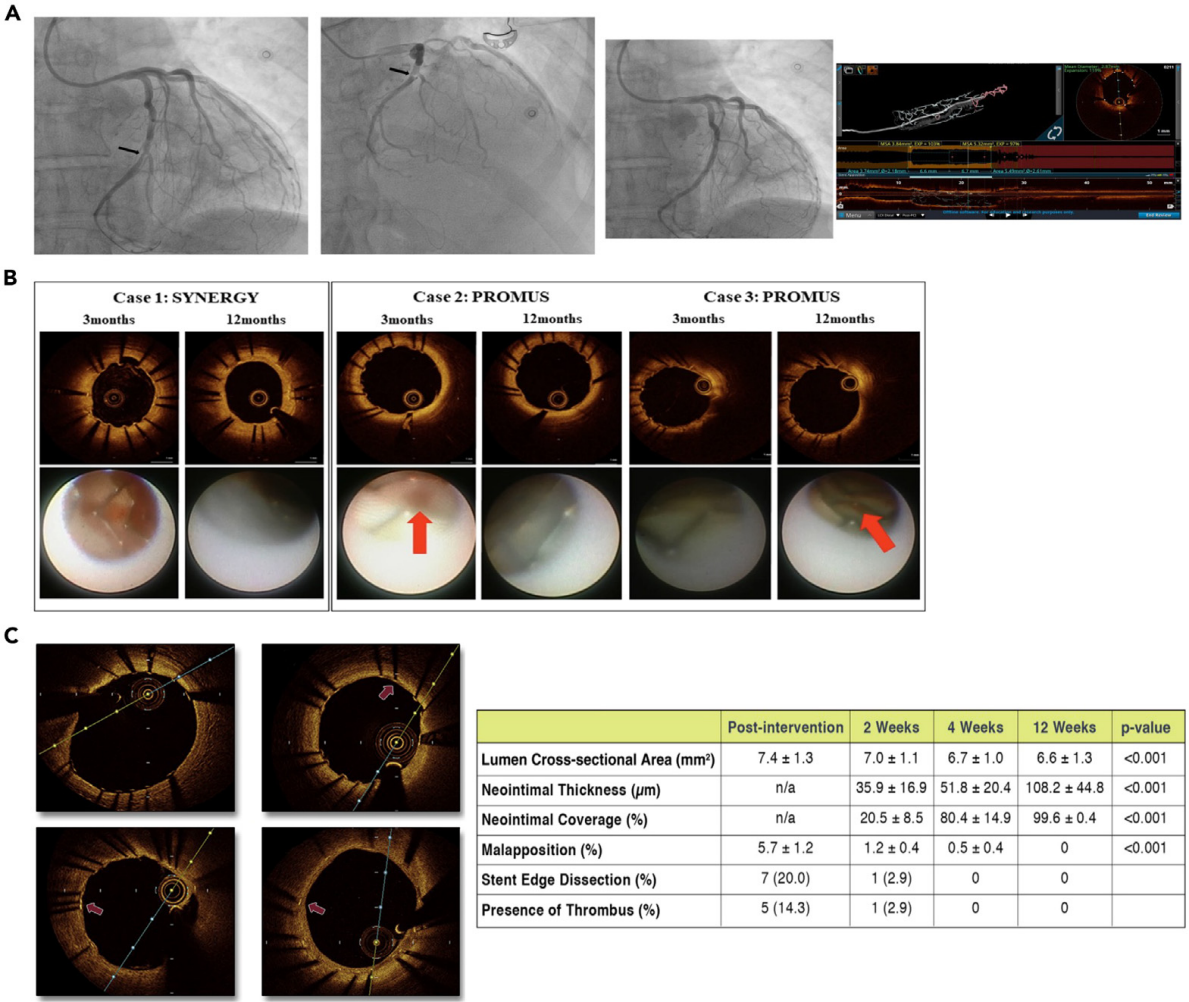
OCT-guided coronary intervention (A) Left coronary artery angiogram and an OCT scan before/after PCI. Reproduced with permission from.^38^ Copyright 2023 Frontiers. (B) Representative OCT and coronary angioscopy findings. Red arrows: thrombus. Reproduced with permission from.^39^ Copyright 2021 Springer Nature. (C) Representative serial OCT images and data of in-stent neointima. Reproduced with permission from.^40^ Copyright 2017 Elsevier.

Detecting vulnerable plaque may also help to identify patients who benefit from aggressive prevention treatments such as proprotein convertase subtilisin/kexin type 9 (PCSK9) inhibitors or anti-inflammatory therapies.^4^ In the study by Gao et al. in 2021, the impact of the PCSK9 inhibitor alirocumab combined with statin therapy on plaque stability in patients with CAD was examined.^29^ Utilizing OCT, the study assessed characteristics of vulnerable plaques, such as minimum fibrous cap thickness and lipid arc length. The results demonstrated that, compared to standard care, the alirocumab group exhibited a significant increase in minimum fibrous cap thickness and a substantial reduction in lipid arc, indicating a shift toward a more stable plaque phenotype (Figure 1B). These findings highlight the potential role of PCSK9 inhibitors in promoting plaque stabilization and underscore the importance of OCT in evaluating the efficacy of such therapies.

#### OCT-guided percutaneous coronary intervention

OCT can guide percutaneous coronary interventions (PCI) by facilitating stent selection, positioning, and deployment, as well as assessing stent apposition and healing.^32^ A 2016 prospective study comparing OCT-guided PCI with IVUS-guided PCI showed that the OCT-guided group had shorter implanted stent lengths and larger post-procedural stent areas, suggesting that OCT guidance can help standardize and streamline PCI procedures.^33^ OCT also has important reference value for stent size selection. Kumar et al. reported a left main disease case where OCT revealed thrombotic lesions in left anterior descending (LAD).^34^ Similarly, an elderly woman with prior vein graft stenting who had recurrent MI was presented.^35^ OCT uncovered thrombus-like lesions and ruptured fibrous cap in the vein graft. Stent under-expansion and malposition detected by post-procedure OCT was corrected by further post-dilation. Taken together, these cases highlight the value of OCT in guiding selection of appropriate stent sizes for optimal implantation results.

Moreover, OCT can be used for immediate evaluation after the stent implantation. In 2019, OCT-guided PCI optimization strategy was prospectively evaluated.^36^ It shows that OCT can identify issues such as stent under expansion, residual stenosis, and edge dissection, guiding timely treatment. Similarly, research by Kang et al. in 2023 confirmed that OCT-guided PCI was noninferior to IVUS-guided PCI with respect to the incidence of a composite of death from cardiac causes, target vessel-related MI, or ischemia-driven target-vessel revascularization at one year.^37^

Recently, Borzanovic et al. reported a case of a 42-year-old male patient who experienced left circumflex (Cx) coronary artery injury after minimally invasive mitral valve repair in 2023.^38^ Post-surgery, the patient developed a non-ST-segment elevation MI. Repeated coronary angiography and OCT examination revealed stenosis in the distal segment of the dominant Cx, with the formation of an intramural hematoma (Figure 2A). OCT clearly identified the injury mechanism as a puncture of the Cx by the mitral annuloplasty sutures, ruling out suture entanglement or external compression. OCT-guided percutaneous coronary intervention achieved good short-term efficacy, highlighting the importance of intraoperative OCT in clarifying coronary injury mechanisms and guiding PCI strategy.

**Figure 3:**
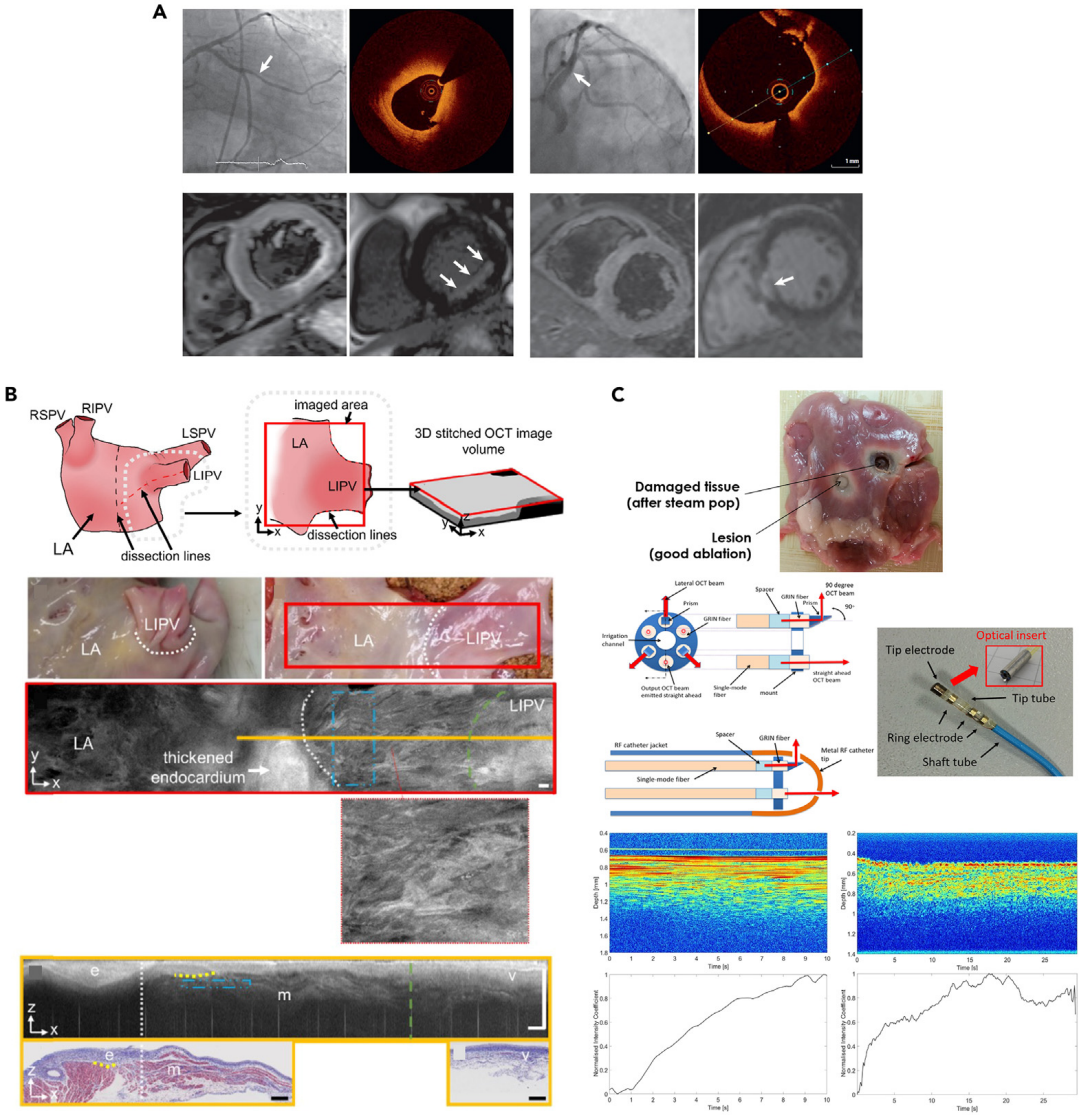
Diagnosis and treatment of myocardial diseases with OCT visualization (A) Coronary angiography/OCT/CMR result patients with non-ST-segment MI. Reproduced with permission from.^45^ Copyright 2019 Elsevier. (B) OCT imaging of a venoatrial junction and corresponding histology, showing changes in endocardial thickness and depth penetration, as well as fibrosis. Reproduced with permission from.^43^ Copyright 2019 Optical Society of America. (C) Fully integrated radiofrequency/optical-coherence-tomography irrigated catheter for atrial fibrillation ablation. Reproduced with permission from.^46^ Copyright 2020 Wiley-VCH GmbH.

#### Application of OCT in follow-up after coronary stent implantation

With the advantage of high-resolution imaging, OCT plays an important role in the follow-up evaluation after coronary stent implantation. Studies have shown that OCT assessment of neointimal hyperplasia, stent coverage and apposition can help guide stent size selection and optimize stent implantation outcomes.^39^^,^^40^^,^^41^^,^^42^ Sakuma et al. compared the vascular healing after SYNERGY vs. PROMUS PREMIER stent implantation in patients with acute coronary syndrome.^39^ The results showed that the SYNERGY group had more significant increase in endothelial progenitor cells at 7 days, and thicker and more complete neointimal coverage by OCT at 12 months, suggesting potential advantages of SYNERGY stent in promoting vascular healing compared with PROMUS PREMIER (Figure 2B). Yamakami et al. used OCT to compare the long-term vascular responses between bioabsorbable-polymer drug-eluting stents (BP-DES) and durable-polymer drug-eluting stents (DP-DES).^41^ In addition, Yano et al. observed that at 12 weeks after SYNERGY stent implantation, OCT showed significantly increased neointimal thickness and nearly complete stent endothelialization, and coronary angioscopy also revealed greater extent of white neointimal coverage on the stent surface compared with control PROMUS stent (Figure 2C).^40^ These studies demonstrate the ability of OCT to clearly depict the quantitative and qualitative features of in-stent neointima, providing new insights for optimizing the design of drug-eluting stents.

### OCT in myocardial disorders

OCT provides high-resolution imaging of myocardial tissue microstructure, showing unique advantages in guiding cardiac surgery and investigating the pathogenesis of myocardial diseases. Current applications of OCT in myocardial disorders mainly focus on two aspects: analyzing myocardial histology and function, and guiding ablation therapy for atrial fibrillation. Regarding myocardial histological analysis and functional assessment, high-resolution OCT enables quantitative analysis of important structural features such as myocardial fiber orientation, density, and collagen fiber distribution, which are closely related to myocardial electrophysiological properties and may influence the occurrence of arrhythmias.^43^^,^^44^ Opolski et al. conducted a meaningful study combining OCT with CMR to evaluate the etiology of MI with nonobstructive coronary arteries (MINOCA).^45^ The study found that 24% of MINOCA patients had plaque rupture detected by OCT, and 18% had coronary thrombus, with these changes more common in the culprit arteries supplying regions of ischemic injury on CMR (Figure 3A). This suggests that atherosclerotic plaque rupture/erosion is an important cause of MINOCA, demonstrating the value of OCT combined with CMR in elucidating MINOCA pathogenesis.

**Figure 4:**
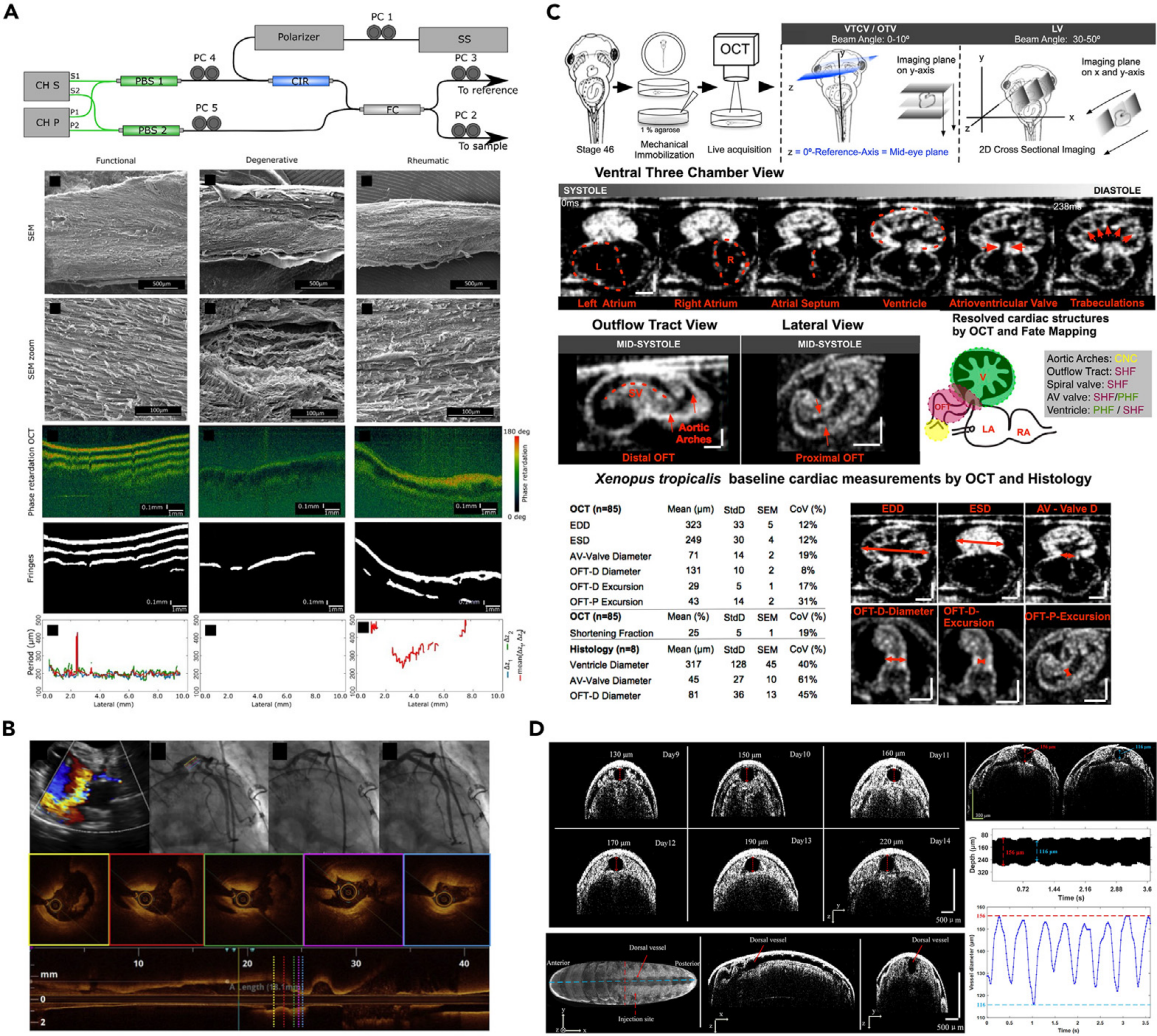
Investigation of other CVDs using OCT (A) Identification of human pathological mitral chordae tendineae using polarization-sensitive OCT (PSOCT-1300). Reproduced with permission from.^52^ Copyright 2019 MDPI. (B) Coronary injury in mitral and aortic valve surgery: direct vessel injury with intimal laceration and thrombus formation. Reproduced with permission from.^53^ Copyright 2020 Elsevier. (C) Imaging the xenopus tropicalis heart with OCT. Reproduced with permission from.^54^ Copyright 2017 Springer Nature. (D) Non-invasive examination of the cardiac properties of insect embryos enabled by OCT. Reproduced with permission from.^55^ Copyright 2022 Wiley-VCH GmbH.

OCT enables high-resolution visualization of the atrial myocardium and pulmonary veins, offering insights into the structural basis of atrial fibrillation. In 2019, Lye et al. pioneered the use of OCT to image the human pulmonary vein-atrial junction, clearly visualizing the distribution of venous endothelium, myocardial sleeves, and fibrosis, providing a morphological basis for OCT-guided atrial fibrillation ablation (Figure 3B).^43^ By comparing the 3D ROI statistics for the extracted texture and fiber orientations in the blue boxes in B, the mean local range of the myocardial ROI is 4.88, the mean local standard deviation is 1.65, and the mean local entropy is 2.89. The circular standard deviation of the extracted fiber orientation angle is 35.17°. Subsequent studies further confirmed that integrating OCT into radiofrequency ablation catheters allows real-time monitoring of lesion formation, predicting steam pops, and reflecting the contact state and angle between the catheter and tissue, thereby avoiding complications and improving procedural safety and efficacy (Figure 3C).^46^ These studies demonstrate the broad application prospects of OCT in atrial fibrillation ablation.

Moreover, OCT can differentiate epicardial adipose tissue and visualize subendocardial fibrosis and atherosclerotic plaques, pathological changes often present in MI and cardiomyopathies.^47^ Another meaningful study found that OCT can also reflect the distribution, density, and diameter of myocardial micro vessels, parameters that can assess myocardial perfusion status and may be abnormal in diseases like heart failure.^48^ Gan et al. performed comprehensive OCT imaging of 50 human heart specimens, detailing the typical features of myocardial tissue, adipose tissue, and fibrosis on OCT. Quantitative analysis revealed significant differences in attenuation coefficients between atria and ventricles, as well as clear distinctions between adipose and other tissue types.^49^ This study provides an important morphological reference for OCT assessment of myocardial tissue.

### OCT in other cardiovascular diseases

#### OCT in heart valve disease

In 2016, Lee et al. used high-resolution OCT for real-time imaging of the aortic and mitral valves in a mouse model of Marfan syndrome,^50^ indicating that OCT can be applied to basic research of animal heart valve disease (HVD). In 2019, Courchaine et al. used polarization-sensitive OCT (PS-OCT, Thorlabs Telesto III) to achieve non-invasive, real-time, dynamic imaging of the endocardial-to-mesenchymal transition (EndMT) process in the outflow tract cushions of chicken embryonic hearts for the first time.^51^ The OCT system employed a central wavelength of 1300 nm, an A-scan rate of 76 kHz, and acquired 580 A-scans per B-scan images with a resolution of 2.68 μm/pixel. During EndMT, endothelial cells migrate into the cardiac matrix to form valve leaflets. The OCT system’s 1–2 mm penetration depth allowed clear distinction of the endothelial, matrix, and muscle layers in the outflow tract. This study offers a novel approach for *in vivo* investigations of EndMT regulatory mechanisms and factors causing cardiac malformations, providing valuable insights through quantitative analysis of dynamic cell population changes within the endocardial cushions during this crucial developmental stage.

The mitral valve consists of leaflets and chordae tendineae (CT). CTs connect the leaflets to the papillary muscles and provide support during valve opening and closing. CT pathologies can lead to mitral valve dysfunction. In 2019, Real et al. used PS-OCT (OCS1300SS, Thorlabs, Newton, NJ, USA) to study the birefringence properties of human mitral valve CTs.^52^ The system provided B-scans with a lateral scan size of 10 mm in length and a penetration depth of 3 mm in air, achieving a resolution of 25 μm laterally and 12 μm axially in air. C-scans with lateral dimensions of 10 mm × 10 mm was obtained, covering the typical length and thickness of CT. By comparing scanning electron microscopy (SEM) and PS-OCT results, a correlation between CT birefringence and collagen structural disorder was confirmed. The study proposed that the birefringence properties detected by PS-OCT can serve as a marker to differentiate normal and pathological CTs and guide surgical treatment decisions (Figure 4A). Alterations in the collagen structure of CTs may be associated with a reduction or suppression of fringes caused by birefringence in OCT, whereas the absence of this parameter indicates the presence of pathological disease. Later, in 2020, Scarsini et al. reported 3 cases of acute coronary artery complications during mitral valve surgery.^53^ OCT examination revealed that coronary deformation, twisting, compression or rupture caused by surgical sutures were the causes of acute ischemia (Figure 4B). This further demonstrates the application value of OCT in mitral valve surgery.

**Figure 5:**
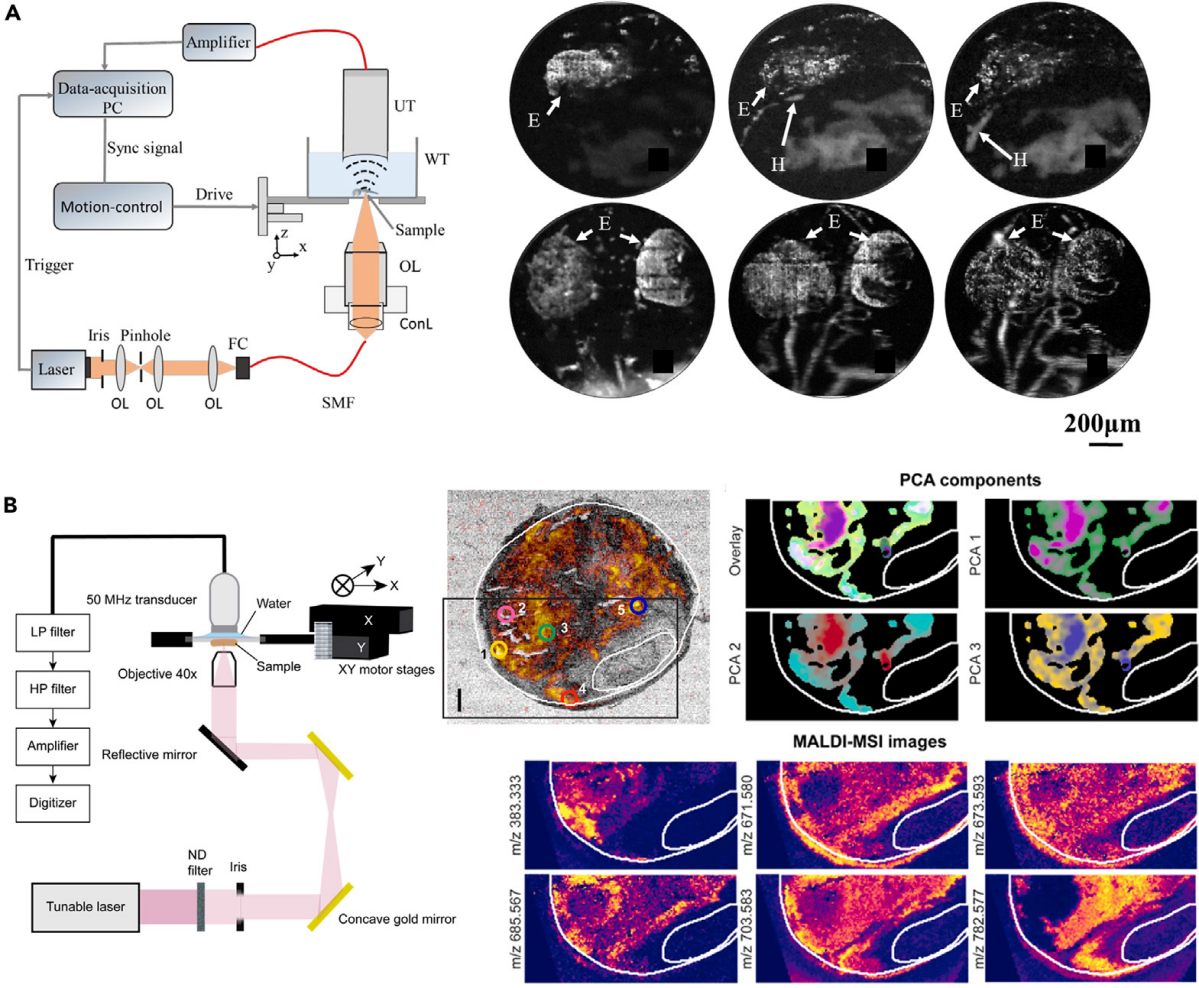
Representative PAM systems for heart detection (A) Left: Schematic of a typical OR-PAM setup. OL: objective; FC: fiber coupler; SMF: single-mode-fiber; ConL: convex lens; WT: water tank; UT: ultrasonic transducer. Right: The imaged vascular development in the heart and brain regions of the zebrafish embryo. H: Heart; E: Eye. Reproduced with permission from.^72^ Copyright 2017 Optical Society of America. (B) (Left) Experimental setup of micro spectroscopic photoacoustic microscopy (μsPAM). (Right) Full field of view image of plaque and lumen at 1210 nm and spatial distribution of 3 principal components of simples P1-2. Reproduced with permission from.^73^ Copyright 2021 Elsevier GmbH.

In summary, OCT technology has broad application prospects in HVD and can be used for research at different levels such as animal models, embryonic development, and human tissues. It plays a unique role in disease diagnosis, basic research, and surgical guidance and is expected to further promote precision diagnosis and treatment of HVD.

#### OCT in congenital heart disease

Congenital heart disease (CHD) is the most common type of congenital malformation in newborns. OCT has shown promising application prospects in evaluating pulmonary artery wall changes in patients with CHD due to its high-resolution imaging capability. In 2018, Homma et al. used OCT to assess pulmonary artery wall changes in 39 pediatric patients with left-to-right shunt CHD.^56^ Regardless of differences in age, body size, or hemodynamics, vessels with diameters between 2 and 3 mm were most likely to show pulmonary artery walls. This study first clinically confirmed that OCT can evaluate the relationship between pulmonary artery wall changes and pulmonary hemodynamics in children with CHD.

OCT has also been applied in animal models of CHD. In 2017, Deniz et al. established various CHD models in Xenopus embryos induced by teratogenic factors and used OCT to analyze the morphology and function of the embryonic hearts, clearly showing structures such as the atrium, ventricle, atrial septum, ventricular septum, and blood vessels (Figure 4C).^54^ Then, in 2022, Su et al. used OCT to non-invasively detect the heart rate, cardiac cycle, systolic/diastolic diameter and other parameters of locust embryos at different developmental stages and temperatures.^55^ The study found that the heart rate of locust embryos increased with developmental progress and temperature, and the heart rate of embryos that experienced a quiescence period was 20% higher than those that did not (Figure 4D). This study applied OCT to evaluate the cardiac activity and gene regulation of insect embryos for the first time, providing new ideas for using invertebrate animal models to study human CVDs.

### Algorithms-based OCT image analysis in CVDs

With the continuous advancement of OCT technology, its application in the diagnosis and treatment of CVDs has become increasingly extensive. In recent years, the integration of AI algorithms has further enhanced the performance and applicability of OCT technology. This opens new avenues for the precise diagnosis and treatment of CVDs.

Deep learning algorithms such as convolutional neural networks (CNNs) have made significant progress in the field of medical image analysis. For instance, applying CNNs to the automatic analysis of coronary OCT images has potentials to improve efficiency and accuracy.^57^^,^^58^^,^^59^^,^^60^^,^^61^^,^^62^ In 2019, Gessert et al. developed an OCT image segmentation algorithm based on CNN that can automatically identify and delineate the contours of coronary artery plaques, enabling quantitative plaque analysis.^57^ In 2020, Lee et al. proposed a hybrid learning strategy which combined CNN and hand-crafted, lumen morphological features to automatically classify and quantitatively assess various tissue types, such as fibro-lipid plaques and fibro-calcific plaques.^59^ Relying solely on features automatically extracted through deep learning, Abdolmanafi et al. employed a more sophisticated deep learning model based on an encoder-decoder architecture, capable of identifying and characterizing a broader range of plaque components in atherosclerotic OCT images.^60^ Furthermore, in 2023, Huang et al. proposed a machine learning algorithm based on OCT angiography (OCTA) that can identify systemic CVDs and metabolic risk factors from ocular OCTA images.^62^ Two models (CNN and MoblieNetV2) achieved AUCs of 0.74 and 0.81 for predicting hyperlipidemia on 3 × 3 mm OCTA images, both with an accuracy of 0.79, demonstrating the potential of using OCTA to screen for cardiovascular and metabolic risk factors.

Other learning architectures have also been investigated for use in OCT image processing in addition to CNN. In 2023, Zafar et al. utilized conditional generative adversarial networks (cGANs) for data augmentation on a newly created intracoronary OCT image dataset to improve the performance of deep learning-based coronary plaque characterization.^63^ They demonstrated that augmenting the original dataset with cGAN-generated synthetic images by a factor of 50x during training enhanced the classification accuracy of the AlexNet model by 15.8%. This study confirms that data enhancement using cGANs can significantly improve the performance of deep learning models when dealing with limited datasets, especially in the recognition and classification of coronary plaques.

Transformer architectures have also played an effective role. For example, intravascular optical coherence tomography (IVOCT) has garnered significant attention in CVD diagnosis due to its high resolution and excellent ability to identify atherosclerotic plaques. However, the presence of noise, artifacts, and the complex morphology of plaques in OCT images often poses challenges for traditional CNN models to accurately segment the plaque regions. Among various models, the transformer has demonstrated superior performance in multiple computer vision tasks, owing to its powerful capabilities in contextual understanding and feature extraction.^64^^,^^65^ Compared to CNNs, transformer models can better capture global information and long-range dependencies within images, thereby enhancing the model’s comprehension of complex scenes.^66^ Recent studies have indicated that employing transformer models can effectively overcome these difficulties and improve the performance of automatic plaque segmentation in OCT images. In 2022, Park et al. developed a transformer-based pyramid network for the diagnosis of coronary plaque erosion, which significantly outperformed existing CNN methods.^64^ This model utilizes a CSWin Transformer as the encoder and incorporates an augmented feature split module and a convolutional position encoding mechanism to enhance the transformer’s ability to capture fine-grained features and global contexts. Then in 2024, Liu et al. proposed a novel transformer encoder-decoder architecture called AFS-TPNet for end-to-end semantic segmentation of plaques in OCT images.^67^ This method embeds an improved transformer backbone into a feature pyramid and designs an augmented feature split module, substantially boosting the model’s ability to extract features at different scales. Moreover, the introduction of a residual convolutional position encoding mechanism enables the transformer to better perceive the positional information of target regions. Experimental results confirm that AFS-TPNet excels in identifying calcified plaques of various shapes and contexts, significantly surpassing existing CNN and transformer architectures.

## Photoacoustic imaging in cardiovascular diseases

The high resolution and *in vivo* biopsies capability of OCT pose a crucial role in the diagnosis and treatment of CVDs. However, its ability is limited in penetration depth due to the sensitivity of photon scattering within tissues, and the lack of microvascular visualization and molecular targeting capabilities make it insufficient for functional diagnosis. Therefore, there is an urgent need for a structural-functional imaging modality with greater depth of penetration and desirable resolution. PAI, also known as optoacoustic imaging (OAI), is a relatively new biomedical imaging approach that uses light-induced ultrasound to examine tissue structure and functionality information.^68^^,^^69^^,^^70^^,^^71^ It has recently been widely recognized as a novel optically relevant imaging technique that allows high-resolution optical contrast imaging at depths ranging from a few millimeters to a few centimeters. The foundation of PAI is the photoacoustic effect, which is produced when tissues absorb pulsed laser energy and expand thermoelastically, which leads to ultrasonic vibrations. Then, the sound waves are collected up by surrounding ultrasound transducers and reconstructed into finely detailed images with absorption distribution in tissues.

Depending on the application scenarios, PAI systems can be roughly categorized into three sorts - photoacoustic microscopy (PAM), photoacoustic computed tomography (PAT/PACT), and photoacoustic endoscopy (PAE), each tailored for specific application scenarios such as surface-level tissue imaging, small animal whole-body imaging, and intracavitary probing, respectively. These systems have been widely applied in various fields, including CVDs. In this section, a comprehensive overview of PAI in cardiovascular applications will be discussed, covering the development of imaging systems, novel agents for different heart diseases, as well as advanced algorithms and learning frameworks.

### Development of photoacoustic systems for cardiac imaging

#### Photoacoustic microscopy

PAM can be further divided into optical resolution PAM (OR-PAM) and acoustic resolution PAM (AR-PAM) depending on whether light is focused on the regions of interest. OR-PAM offers high spatial resolution, but functions only at superficial depths within a few hundred micrometers beneath skin, while AR-PAM can penetrate deeper into tissue as it does no rely on focused light delivery, but at the cost of spatial resolution. OR-PAM is preferred more for cardiac imaging applications. For example, Chen et al. developed a label-free OR-PAM system using a 50 MHz transducer to image the cardio-cerebrovascular development in the embryonic zebrafish,^72^ as shown in Figure 5A. The formation of the cardiovascular network was successfully observed at a lateral resolution of 1.5 μm with a 10× objective, and the development of blood vessels throughout the body was monitored at a lateral resolution of 3.5 μm with a 4× objective. However, for a whole-body scan, imaging took about 25 min, a rate that is insufficient to observe real-time changes in the living heart. In another study, Iskander-Rizk et al. used OR-PAM and matrix-assisted laser desorption ionization mass spectrometry imaging to extract plaque lipid PA spectral features of human endarterectomy samples in the wavelength range 1150–1240 nm,^73^ as depicted in Figure 5B. Differences in the spectral characteristics of lipids extracted by this method revealed three distinct components with peaks at 1164, 1188, 1196, and 1210 nm, respectively. This OR-PAM based spectral separation technique provides guidance for atherosclerotic disease staging. Note, however, this implementation can thus far be used only for tissue slices, but not live bodies.

**Figure 6:**
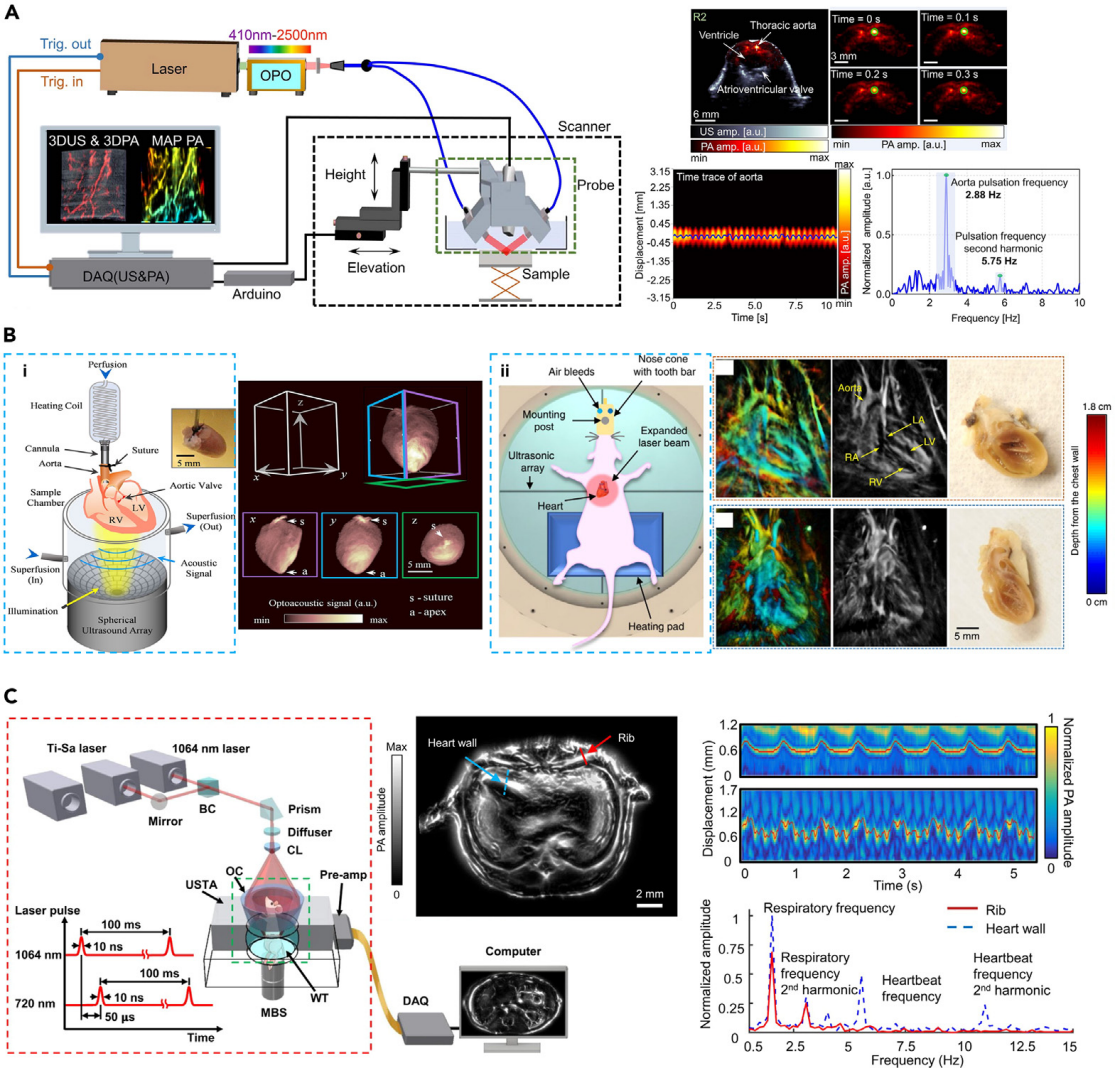
Development of PAT/PACT system in heart visualization (A) Video-rate WBHUS/PA dual-modal imaging platform, overlaying harmonic US and PA images at the cross-section of the heart and the heartbeat analysis. Reproduced with permission from.^75^ Copyright 2022 IEEE. (B-i) Imaging setup of real-time volumetric optoacoustic imaging of Langendorff-perfused heart and cross-sectional images of the whole Langendorff-perfused heart. Reproduced with permission from.^76^ Copyright 2018 Springer Nature. (B-ii) Experimental setup for the rat heart imaging and differences in cardiac anatomy and function between the Zucker obese and lean rats. Reproduced with permission from.^77^ Copyright 2023 Springer Nature. (C) Schematics of the SIP-PACT system for trunk imaging and label-free imaging of small-animal heart dynamics. Reproduced with permission from.^78^ Copyright 2017 Springer Nature.

#### Photoacoustic computed tomography

Depending on the arrangement of ultrasound transducer, PAT or PACT can be further divided into linear-, hemispherical-, spherical-, and ring-array types. For example, Zemp et al. improved a PA imaging system based on a fast pulsed laser and a high-frequency ultrasound array transducer, and reported its application to high-speed imaging of the beating heart in mice,^74^ which is the first publicly available research on real-time PA imaging of physiological dynamics. PACT can easily share the linear-array ultrasound detectors with clinic ultrasound devices, which has expanded its clinical applications. For example, Zhang et al. established a wide-beam harmonic ultrasound and PAT (WBHUS/PA) imaging system at a video frame rate. This wide-beam ultrasound transmission can reduce the scan time by 267% and achieve an imaging rate of up to 20 Hz, minimizing motion artifacts in live imaging.^75^ Such performance allows real-time access to the structure of the heart and parameters such as heartbeat and respiratory rate through heartbeat cycle monitoring, thus reflecting dynamic cardiovascular changes (Figure 6A).

**Figure 7:**
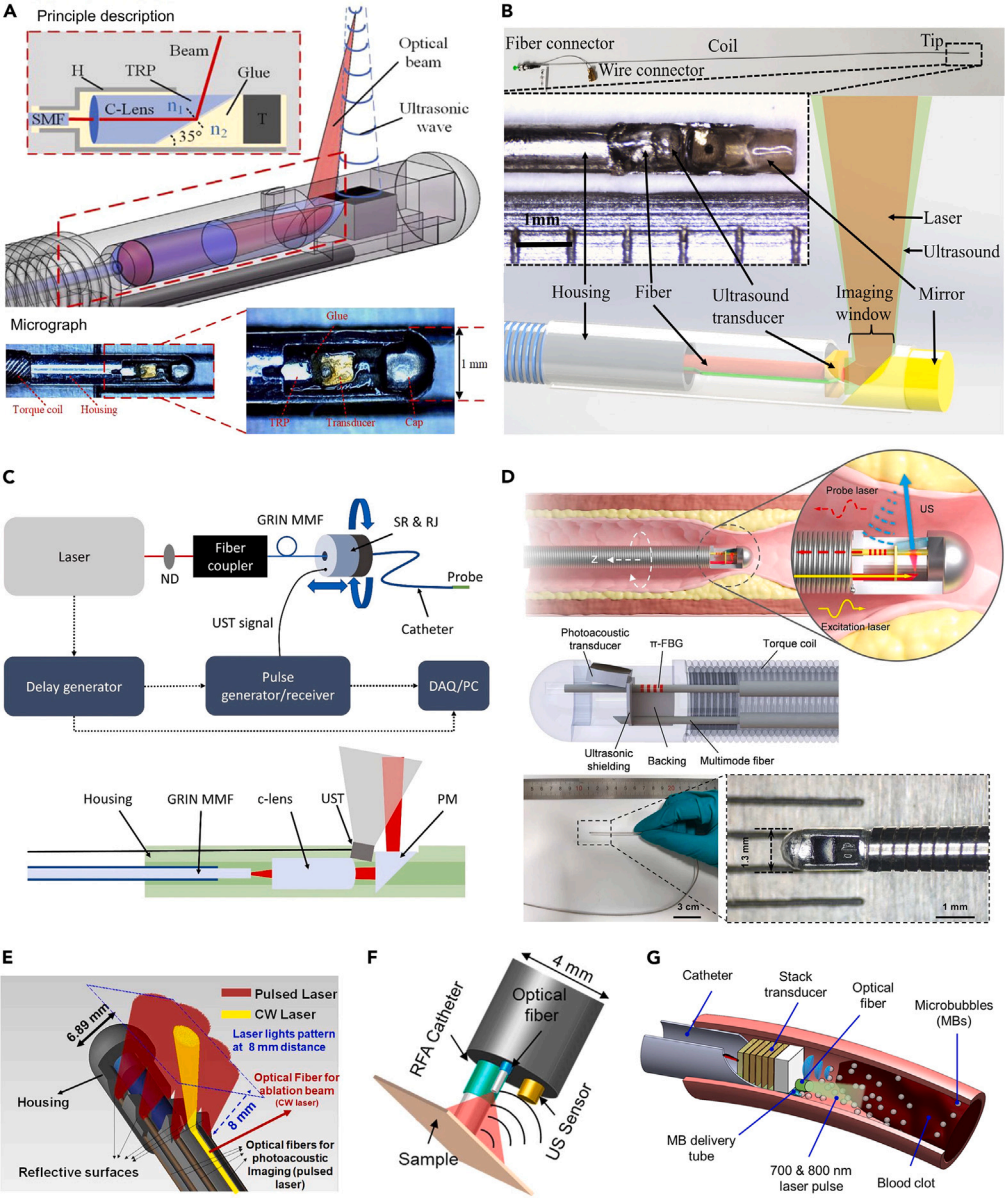
Development of IVPA for detection and ablation monitoring of CVDs (A) Design and assembly of the HR-IVPA endoscope with hermetically sealed opto-acoustic capsule. Reproduced with permission from.^83^ Copyright 2020 Optical Society of America. (B) Photos and schematic of the coaxial IVPA catheter. Reproduced with permission from.^84^ Copyright 2022 Wiley-VCH GmbH. (C) Schematic of the IVPA imaging system and probe. Reproduced with permission from.^85^ Copyright 2023 Elsevier GmbH. (D) AO-IVUS imaging system and catheter. Reproduced with permission from.^86^ Copyright 2023 Science. (E) A schematic of miniaturized integrated US/PA-guided laser ablation theranostic system. Reproduced with permission from.^87^ Copyright 2021 IEEE. (F) Design of the proposed miniaturized catheter-integrated PA RFA ablation system. Reproduced with permission from.^88^ Copyright 2022 IEEE. (G) Interior design of the two-lumen catheter for the sonothrombolysis. The miniaturized stacked transducer was mounted in the main lumen with a side lumen for the microbubble (MB) delivery and laser light delivery. Reproduced with permission from.^89^ Copyright 2021 IEEE.

The hemispherical detector-based PACT system has been used for breast detection in clinics.^79^^,^^80^^,^^81^ Later, researchers found that it could also be used for real-time cardiac monitoring. For example, Lin et al. developed a fast volumetric optoacoustic tomography platform for functional imaging of the entire isolated Langendorff perfusion heart using a spherical-matrix array transducer.^76^ They demonstrated imaging of deep cardiac features, including the interventricular septum, chordae tendineae, and papillary muscles, while further tracking in real time the cardiac beat cycle as well as pulmonary, mitral, and tricuspid valve motion. This work enabled the first four-dimensional imaging of the entire beating isolated mouse heart with PA methods (Figure 6B-i). In 2024, the same team advanced this system for whole-heart visualization of living mouse embryonic model, with high spatial (100 μm) and temporal (10 ms) resolutions.^82^ They tracked the development of the embryonic heart at 14.5–17.5 days of gestation, and the spectral recordings allowed for the quantification of blood oxygenation within the cardiac chambers in a labelling-free, non-invasive manner. This technique offers new possibilities for high-resolution quantification of embryonic cardiac function at different gestational stages in mammalian models, providing a valuable non-invasive method for developmental biology. Lin et al. developed a 3D PACT platform to achieved noninvasive imaging of rat hearts without surgical procedure (thoracotomy) for tissue penetration.^77^ They found that the platform enabled unobstructed visualization of cardiac anatomy and intracardiac hemodynamics (Figure 6B-ii). Further, they revealed different structural and functional changes in the heart between healthy, hypertensive, and obese rats, with optical comparisons reflecting the differences in chamber size, wall thickness, and hemodynamics.

The hemispherical-array PACT system still has some drawbacks like an effective scanning angle of merely 140°,^76^ which leads to partial loss of signals and lower detection sensitivity. To overcome these, researchers have proposed a PACT system based on a ring-array ultrasound transducer, which can guarantee a full 360°-field-of-view scan. A single-impulse panoramic photoacoustic computed tomography (SIP-PACT) was developed,^78^ which has combined all advantages of high temporal and spatial resolution (125 μm in-plane resolution, 50 μs/frame data acquisition, and 50 Hz frame rate), deep penetration (*in vivo* cross-sectional width of 48 mm), anatomical, dynamic, and functional contrasts, and full-view fidelity. The authors imaged the whole-body dynamics of small animals in real time and obtained clear anatomical and functional details of sub-organs, including the heart. By extracting the position of the dynamic heart wall and analyzing the frequency, important parameters such as respiratory rate and heartbeat can be obtained (Figure 6C).

#### Intravascular photoacoustic endoscope

Endovascular heart diseases such as atherosclerotic cardiovascular disease (ASCVD) account for a high proportion of morbidity and mortality worldwide. Intravascular photoacoustic endoscope (IVPAE) imaging is rapidly emerging as a novel imaging solution for the diagnosis and treatment of ASCVD. Compared with IVUS and OCT, IVPAE provides structural, functional, and molecular information of biological tissues with high spatial resolution, deep penetration, and lipid plaque-specific identification. For example, an opto-acoustic capsule that was hermetically sealed was designed to create a high-robustness intravascular photoacoustic (HR-IVPA) endoscope.^83^ In this endoscope, the laser beam was transmitted through a single mode fiber, focused by a C-lens, and ultimately re-reflected by a total reflecting prism (TRP). The TRP was first applied to side-viewing IVPAE with 90% high-throughput energy coupling characteristics and a no-cut damage threshold. The air gap of the capsule was filled with an index-matched optical glue for all solid seals and total reflection (Figure 7A). With an external dimension of only 1 mm, a resolution of 23 μm and a signal-to-noise ratio of 41.8 dB were achieved in arterial lumen experiments. Recently, another acousto-optic coaxial design of the IVPAE was developed.^84^ It had a diameter of only 0.9 mm. Within the unit, a miniature ultrasound transducer of 0.18 mm in diameter was arranged, with an orifice in the center to allow the laser light to pass through the orifice. Light was then reflected by a mirror at the distal end into preferred tissue regions. The generated PA signals were coaxially reflected and received by the ultrasound transducer (Figure 7B).

**Figure 8:**
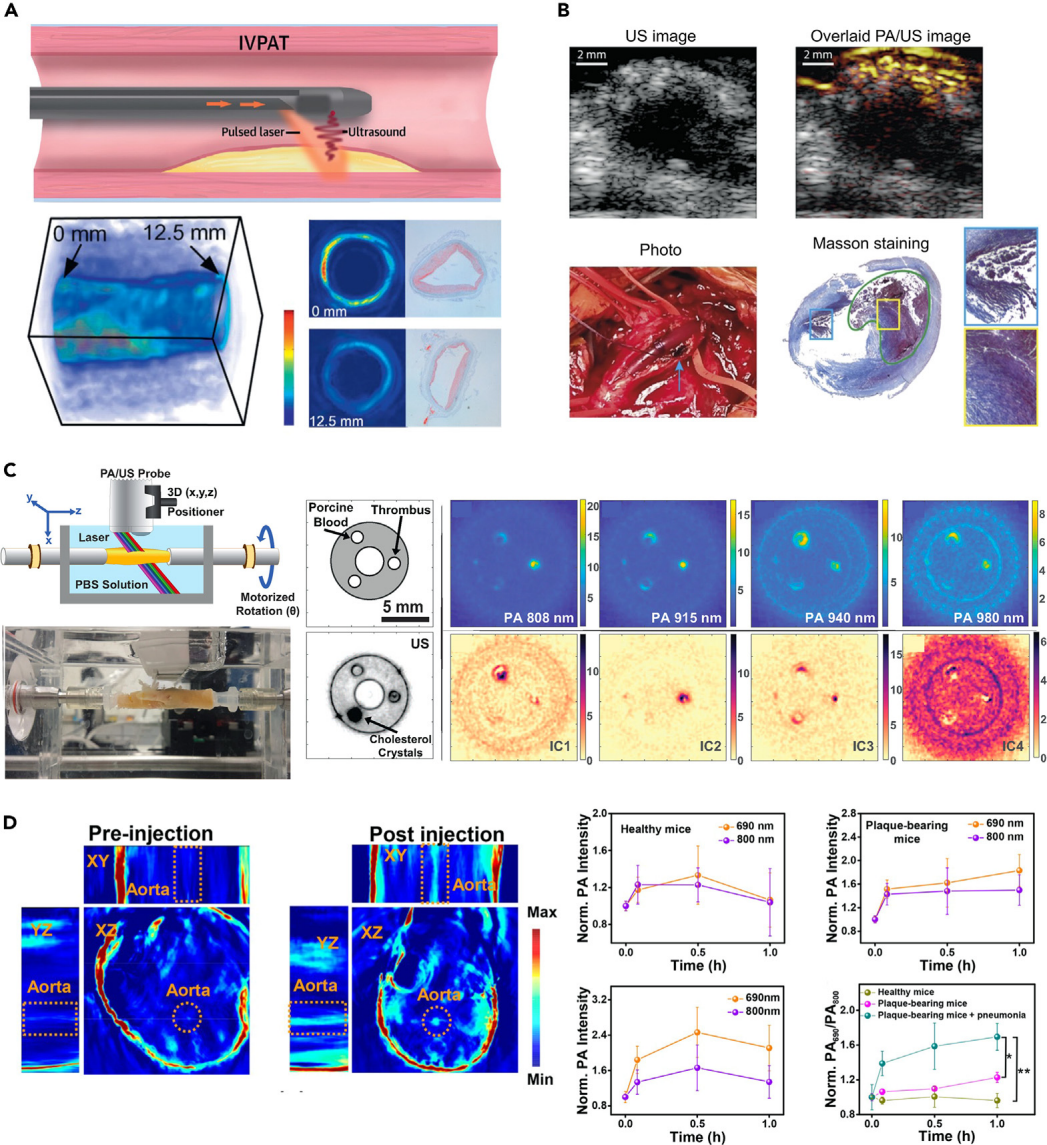
Applications of PAI in CADs (A) The design of IVPAT catheter, and 3D Imaging of lipid within intact atherosclerotic aortas. Reproduced with permission from.^98^ Copyright 2014 Elsevier Inc. (B) *In vivo* PA and US image of a human carotid artery with intraplaque hemorrhage. PA at 808 nm, dynamic range of 23 dB. Reproduced with permission from.^99^ Copyright 2021 Optical Society of America. (C) Illustration and picture of the experimental setup and a cross-section image of PVA phantom. Reproduced with permission from.^100^ Copyright 2019 Elsevier GmbH. (D) 3D PA image of plaque-bearing mice before and after injection of RSPN. Excitation: 690 nm. Aorta regions were depicted by dotted circles. Normalized PA690, PA800 and PA690/PA800 ratios of different mice modes. Reproduced with permission from.^101^ Copyright 2021 American Chemical Society.

Considering that fact that lower order modes generated by the self-cleaning effect in the graded index fiber greatly improved the spatial resolution and detection sensitivity of PAI compared to the high order modes, Yuan et al. developed an IVPA catheter with mode self-cleaning in a graded index multimode fiber to improve the light beam quality and detection sensitivity of lipids (Figure 7C).^85^ The imaging was evaluated on a model simulating lipid plaque, and the results showed that lipid particles with a small diameter of 75.7 μm could be clearly observed. In another interesting design, Wang et al. reported an all-optical intravascular ultrasound (AO-IVUS) imaging system that used picosecond laser pulses to pump carbon composites for ultrasonic excitation and π-phase-shifted fiber Bragg gratings for ultrasonic detection.^86^ Using this all-optical technique, they achieved ultra-wide bandwidth (147%) and high-resolution (18.6 μm) IVUS imaging that was unattainable with conventional techniques. Rotational pullback imaging scans were performed in rabbit iliac arteries and porcine coronary arteries, etc. In comparison with commercial venous ultrasound scans, high-resolution AO-IVUS has advantages in depicting the details of vascular structure and has great potential for clinical applications (Figure 7D).

Currently, ablation is the mainstay treatment for cardiovascular diseases. However, the gold standard of treatment monitoring, usually utilizing computed tomography (CT) or magnetic resonance imaging (MRI) for pre- and post-procedure evaluation, lacks real-time spatial information of the ablation process. On this aspect, PAI could addresses this limitation, allowing the operator to observe the progress of the ablation process. In recent years, combinations of multiple ablation techniques and real-time PA detection have evolved, such as laser ablation,^87^^,^^90^ radiofrequency ablation,^88^ and ultrasound ablation.^89^ For example, Basij et al. developed a US/PA guided laser ablation therapy system.^87^ It consisted of a phased array US probe, six optical fibers for PA imaging, an optical fiber for ablation, and a silver housing with seven reflective surfaces to direct the light toward the tissue (Figure 7E). The overall diameter of the endoscope probe is 6.89 mm. The system allowed real-time monitoring of functional changes and progress at the ablation site. Then, a miniaturized catheter-integrated PA radiofrequency ablation (RFA) monitoring system for treatment and monitoring in a single insertion was proposed.^88^ The authors integrated an ultrasound transducer, fiber optics, and RFA into a 4 mm diameter catheter and evaluated the PA RFA catheter using live porcine cardiac tissue. It was shown that the ablation scope provided temporal feedback over a wide range of PA spectral scans, thus enabling ablation monitoring (Figure 7F). For ultrasound ablation, Wu et al. designed a micro-transducer consisting of an 8-layer PZT-5A stack with an aperture size of 1.4 × 1.4 mm^2^ that was mounted in a custom-made dual-lumen 10-Fr catheter for IVUS thrombolysis.^89^ The treatment process was monitored by internally illuminated photoacoustic tomography (II-PAT) using thin optical fibers integrated with the intravascular catheter for intravascular light delivery (Figure 7G).

#### Robotic and flexible photoacoustic techniques in cardiovascular imaging

Most recently, home healthcare is becoming more and more popular. The development of AI^91^ and flexible electronic technology^92^ provide foundation for portable diagnosis and treatment as well. For example, Zhao et al. designed a carotid artery scanning robot based on PA effect.^93^ It can replace doctor’s manual examination by simply clamping the PA probe that was already in the laboratory to this robot. The simulation results show that the robot can be compatible with existing PA system, thus enabling uniform and low-cost PA imaging capability. In addition, vascular simulation of PAI was performed for experimental validation. The experimental results show that it can display many details and contour information of 3D blood vessels well, providing a good guide for subsequent *in vivo* experiments. Graham et al. proposed the use of a robotic PA system to address radiation risks and the instability of hand-inserted catheters in cardiac interventional procedures.^94^ The system uses the PA-based robotic vision servoing method presented in their former works,^95^^,^^96^ which allows the ultrasound probe to be held by the robot and autonomously centered on the probe based on PA signals from the catheter tip. The reliability of the system was verified under live porcine cardiac surgery and histopathological analysis of the excised heart tissue revealed no laser damage.

Quantitative and multiparametric blood analyses are clinically important in the diagnosis of CVDs. Flexible sensors allow for dermal assessment of a wide range of vital signals, but generally exhibit limited functionality in monitoring blood characteristics. To overcome that, Jin et al. combined PAI with flexible electronics to report a flexible optoacoustic blood “stethoscope” (OBS) for multiparametric cardiovascular monitoring.^97^ It was shown that the OBS can be attached to the skin for continuous, noninvasive, and *in situ* monitoring of a wide range of cardiovascular biomarkers, including hypoxia, attenuation of intravascular endogenous drug concentrations, and hemodynamics, which can be further visualized by customized 3D algorithms. The proposed flexible solution avoids the traditional complex fixture setup and is more flexible and accurate in CVD prediction and diagnosis.

### PAI in coronary artery diseases

Vulnerable atherosclerotic plaques consist of a lipid-rich necrotic core covered by a thin fibrous cap that is made vulnerable by macrophage infiltration. The most common triggers of CAD are rupture of vulnerable plaques and thrombosis. Specific identification of plaque and thrombus progression is therefore an important indicator for the diagnosis of CADs, which is met by PAI’s specific absorption imaging of lipids and blood. Zhang et al. used catheter-based IVPAT for the assessment of pixel-based lipid relative concentration (LRC) in the vessel wall.^98^ IVPAT was performed in rabbits with a high-fat diet (HFD). Cross-sectional LRC maps display information on the concentration and depth of lipid content in atherosclerotic plaques. It was found that lipid accumulation within the plaque correlated with the duration of an HFD as assessed by maximum LRC value, mean LRC value, and high-fat content zone. 3D LRC maps allow a comprehensive assessment of focal plaques in the uninjured aorta, including spatial and structural characteristics (Figure 8A). LRC maps obtained *in vivo* accurately show the structure of the lipid core with high contrast. The above results are sufficient to demonstrate the ability of IVPAT to characterize lipid-rich plaques spatially and quantitatively. In addition, Zhang et al. demonstrated the feasibility of survival IVPA/US imaging of the same lipid plaque in the same animal using a sheathed 0.9-mm IVPA catheter and a rabbit model of aortic balloon injury, aided by *in vivo* MRI.^102^ The reported imaging system and methodology promises to be an asset in understanding the progression of atherosclerosis and evaluating the efficacy of medical therapies.

**Figure 9:**
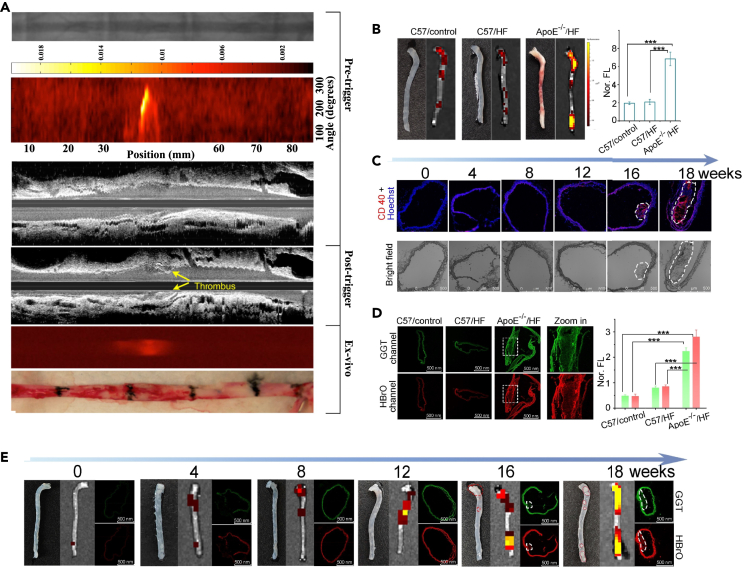
FL imaging of atherothrombosis (A) *In vivo* and *ex vivo* NIR FLI of atherothrombosis. From top to bottom, X-ray angiography mapped the aorta for reference, with subsequent imaging providing a 2D projection of atherosclerotic areas, IVUS imaging before and after pharmacological triggering showed thrombus formation, correlating with regions highlighted in the NIR FLI, further analysis confirmed the *in vivo* findings, and gross pathology was used to examine the aorta and ensure accurate histological correlation. Reproduced with permission from.^137^ Copyright 2017 Wolters Kluwer Health. (B) *Ex vivo* FLI of C-HBrO-GGT in mouse aortas. (C) Confocal FLI of C-HBrO-GGT in frozen aorta sections. (D) Confocal fluorescent images showing CD40 epitopes (red fluorescence) and nucleus (blue fluorescence) with plaques marked by white dotted curves. (E) *In vivo* imaging and confocal imaging of C-HBrO-GGT in mouse aortas at different time points. Reproduced with permission from.^138^ Copyright 2023 Wiley-VCH GmbH.

Meanwhile, PAI allows for real-time monitoring of plaque morphology and analysis of components to assess stenosis and plaque vulnerability, which is essential for intraoperative assessment of endarterectomy. Muller et al. conducted an intraoperative pilot study of carotid plaques in 16 patients using a handheld PAI system.^99^ The results show that PAI is able to detect the presence of hemorrhage and a strong diffuse signal can be observed *in vivo* (Figure 8B). Multispectral photoacoustic imaging (MSPAI) is specific for morphological assessment of carotid plaques. Arabul et al. used MSPAI and non-negative independent component analysis (ICA) to blindly eliminate the confounding of different signal sources in human carotid plaques.^100^ The feasibility of this method was demonstrated on plaque models with hemorrhages and cholesterol inclusions as well as on human samples from plaque endarterectomy (Figure 8C). The results showed that ICA could distinguish new from old hemorrhages. In addition, features of cholesterol inclusion bodies were captured in the model experiments. The unmixed technique could be used for morphological assessment of intact human plaque samples in future preclinical studies.

Note that, however, *in vivo* imaging of PAI generally requires the use of nanoprobes to enhance the signal from the target or the detection contrast because of strong absorption of unlabeled blood. Near-infrared (NIR) nanoprobes typically have excellent optical absorption features, which can greatly improve the sensitivity of PAI even when challenged by strong blood background interference. Therefore, research on targeting probes for CADs has gradually emerged in recent years.^103^^,^^104^ Ge et al. co-assembled osteopontin antibody (OPN Ab), NIR fluorescent molecule indocyanine green (ICG) and Ti_3_C_2_ nanosheets to form nanoprobe OPN Ab/Ti_3_C_2_/ICG.^105^ The designed probe successfully achieved significant NIR-FLI of foam cells and vulnerable plaques with enhanced PA performance, which is effectively for direct and noninvasive *in vivo* imaging of vulnerable atherosclerotic plaques. To accurately assess the risk of plaque rupture due to acute pneumonia, Ma et al. developed a novel ratiometric semiconducting polymer nanoparticle (RSPN) for PA imaging of O_2_^•−^ level within atherosclerotic lesions in mice with pneumonia complicated by apolipoprotein E deficiency.^101^ The RSPN reacts with oxygen and displays enhanced PA signals at ∼690 nm, while 800 nm is considered as an internal PA reference. Thus, the ratiometric PAI (PA690/PA800) by RSPN specifically distinguishes mice with plaques and mice with plaques complicated by pneumonia from healthy mice (Figure 8D). Jiang et al. proposed the use of Trojan foam cells encapsulated with phospholipid dioleoylphosphatidylserine (DOPS), and Cypate-PC, to form functional liposomes (DCP liposomes).^106^ This lipid droplet-hitchhiking strategy enables homologous targeting of atherosclerotic plaques and simultaneous FL/PA imaging. There are other studies that employ plaque site specific targets such as caspase B (CTB) or γ-glutamyl transferase (GGT) to build nanoprobes. For example, Ma et al. developed a lipid-unlocked CTB response probe (L-CRP) for measuring CTB activity in lipophilic environments,^107^ and Wang et al. constructed an HDS-GGT for monitoring the GGT dynamics in CADs.^108^ The diagnostic and therapeutic synergies for blood clots or plaques also have received considerable research attentions that are involved with the use of hydrogen peroxide (H_2_O_2_) activation mechanisms,^109^ multichannel regulation of lipid dissolution and metabolism,^110^^,^^111^ and immune microenvironment rebuilding strategy,^112^ etc.

### PAI in myocardial infarction

MI remains the second largest category of cardiac deaths worldwide. Timely and accurate diagnosis and longitudinal monitoring are essential for optimal patient care. Lv et al. used a hemispherical PAI system to depict the morphology of the thoracic vasculature and the entire heart, showing gradual enlargement of the infarcted area with necrosis and fibrosis by longitudinal observation.^113^ This work demonstrated the ability of PAI for the diagnosis of MI disease. Accurate delineation of MI boundaries using noninvasive techniques is essential for the treatment and prognosis of the disease. Ma et al. used dual wavelength photoacoustic spectral analysis (DWPASA) to calculate the "the ratio of the areas of the power spectral densities (R_APSD_) " of different regions.^114^ Compared with normal tissue, MI tissue contains more collagen and therefore has a higher R_APSD_ value. The MI cross-section lengths and MI region boundaries delineated by DWPASA in both dimensions matched well with the results of Masson staining. The results suggest that the DWPASA method may provide a new diagnostic method for determining the MI boundaries. Fibronectin is an extracellular matrix protein involved after MI. Zhang et al. constructed fibronectin-targeted nanoparticles (CREKA-ICG-LIP NPs) by co-assembling fibronectin-targeting peptide (CREKA) and ICG and used them for enhanced PAI to detect infarcted areas noninvasively and help diagnose MI.^115^ Zhao et al. developed an ultrasmall nanoprobe self-assembled by single-stranded DNA (ssDNA)/metal ion complexes.^116^ After intravenous injection of DNA-Bi2S3 NPs in myocardial ischemia/reperfusion (I/R) mice, PA signals in the infarcted region were significantly enhanced. The results demonstrated that this NPs is an effective PA probe for imaging the infarcted region. Inspired by post-infarction type I and type III collagen accumulation, Li et al. developed a collagen-targeted multimodal imaging nanoplatform, CNA35-GP@NPs, which consists of lipid NPs, encapsulated gold nanorods (GNRs), and perfluoropentane (PFP). The probe’s surface-modified peptide CNA35 has excellent collagen fiber targeting properties, which facilitates US/PA/CT imaging of MI diseases. In an MI model of a rat, CNA35-GP@NPs enabled multimodal imaging of fibrotic myocardium.^117^

Mitochondrial dysfunction and oxidative damage are important pathological mechanisms in I/R injury. Sun et al. designed gold-selenium core-shell nanostructures (AS-I/S NCs) with good NIR-II PAI for the treatment of I/R.^118^ The AS-I/S NCs were modified using ischemic myocardium-targeting peptide and mitochondria-targeting antioxidant peptide SS31 to achieve enhanced cardiomyocyte-targeted uptake and antioxidant capacity, and to achieve inhibition of cardiotoxic apoptosis and oxidative damage in H9c2 cells. The results showed that *in vivo* injection of AS-I/S NCs in rats significantly improved myocardial function and angiogenesis and inhibited myocardial fibrosis. Inspired by the naturally occurring antioxidative allomelanin extracted from fungi, Mo et al. elaborated allomelanin nanoparticles (AMNPs) to enhance the therapeutic efficacy against I/R injury.^119^ These PEGylated AMNPs@PEG nanomedicines possessed excellent abilities to scavenge intracellular free radicals, inhibit depolarization of mitochondrial membrane potential and enhance cell survival. Due to the increased vascular permeability during myocardial I/R injury, AMNPs@PEG can be targeted and aggregated at the site of cardiac damage for PA visualization. Importantly, AMNPs@PEG achieved macrophage polarization from M1 to M2 subtypes and inhibited neutrophil recruitment, while decreasing the expression of pro-inflammatory genes and elevating the expression of anti-inflammatory genes, respectively. These resulted in a significant reduction in myocardial infarct size and significant improvement in cardiac function after I/R injury.

### PAI in other cardiac diseases

Diabetic cardiomyopathy is a common clinical complication, and the study of vascular microcirculatory dysfunction can provide a reliable basis for diagnosis. Loai et al. used PAI to monitor skeletal muscle microvascular dysfunction in a sex-specific rat model of type II diabetes.^120^ The study found that skeletal muscle microvascular dysfunction varied by gender, but preceded significant changes in the heart, such as thinning, fibrosis, or hypertrophy, in both male and female patients. Acute hyperglycemia induces endothelial dysfunction and leads to vasoconstriction, increasing cardiovascular risk. To monitor microvascular changes during acute hyperglycemia, Ahn et al. used high-resolution PAM to noninvasively monitor morphological changes in limb blood vessels of living animals during rapid elevation of blood glucose.^121^ After intraperitoneal administration of glucose, heart/respiratory rate and body temperature remained unchanged as blood glucose levels rose from 100 mg/dL to 400 mg/dL, but arterial vasculature constricted by approximately −5.7 ± 1.1% over 20 min and gradually recovered over 40 min. In contrast, venous diameter remained within approximately 0.6 ± 1.5% during arterial constriction. These results demonstrate that acute hyperglycemia causes transient vasoconstriction of the arterial vasculature, in contrast to the trend in blood glucose.

Noted that rapid PA volumetric imaging can avoid the obstruction of a beating heart and acquire the propagation of electromechanical waves in the heart muscle, which is key for determining arrhythmias and other cardiac diseases. Ozsoy et al. demonstrated that sparse optoacoustic sensing using cardiac volumetric motion enables ultra-fast four-dimensional imaging of cardiac mechanical wave propagation throughout the beating mouse heart.^122^ The method is based on fast compressed acquisition of optoacoustic responses from a random subset of US detection channels, followed by iterative reconstruction of the entire image sequence using the minimum convolution with total variation function. This effectively outlines the multiscale spatiotemporal information encoded by cardiac volumetric motion with high contrast, ∼115 μm spatial resolution, and submillisecond temporal resolution. This cardiac mapping approach provides a powerful tool for deciphering the complex mechanisms of arrhythmias, enabling precise therapeutic interventions.

Cardiac arrest is another common cause of death each year, primarily due to post-cardiac arrest syndrome leading to comprehensive hypoxia and dysfunction of multiple organs after resuscitation. The ability to quantify vascular changes and tissue oxygenation is critical to tailor patient care to minimize major post-resuscitation consequences. Salvas et al. used high-resolution PAI to track trajectories of neurovascular oxygenation and cardiac function in a mouse model of cardiac arrest and resuscitation.^123^ The results showed a higher degree of protection of cerebral oxygenation during cardiac arrest compared to peripheral tissues. They quantified biomarkers such as oxygen saturation, total hemoglobin, overall longitudinal strain of the heart, and ejection fraction to determine the correlation between neurological and cardiovascular parameters that may be useful in predicting functional outcomes from an imaging perspective.

### Algorithms of PAI for cardiac detection

Imaging algorithms are critical to assure the image quality. Associated with the rapid development of PA applications in recent years, we also witnessed a boost of the image reconstruction and enhancement algorithms. PA image reconstruction is generally performed using delay-and-sum (DAS) beamforming techniques. However, non-adaptive DAS reconstructed cardiac PA images are subject to time-varying noise, resulting in reduced myocardial PA specificity. Mukaddim et al. proposed an adaptive beamforming algorithm that extended coherence factor (CF) weighting to the time domain for preclinical cardiac PAI.^124^ The proposed spatiotemporal coherence factor (STCF) takes into account multiple temporally adjacent image acquisition events during beamforming and eliminates external signals with low spatial and temporal coherence, thus removing higher background noise while preserving the main feature of interest (myocardial wall) in the PA images. Later, the same team introduced another adaptive beamforming method, PA sub-aperture processing (PSAP), to mitigate these image degradation effects.^125^ In PSAP, a pair of DAS reconstructed images is formed by splitting the received channel data into two complementary non-overlapping sub-apertures. A weighting matrix is then derived by analyzing the correlation between the sub-aperture beamforming images and multiplied with the full-aperture DAS PA image to reduce residuals and incoherent clutter. Then, the group proposed to use spatiotemporal singular value decomposition (STSVD) processing algorithm^126^ or coupled PSAP+STSVD^127^ to solve the cardiac reconstruction problems. In addition, they proposed a physiological signal gated PAI technique with motion compensation, called OPMC, for subtle oxygen saturation estimation of ischemia blood vessels.^128^

PA image reconstruction is the recovery of absorbed optical energy density (AOED) and optical absorption coefficient (OAC) distributions of vascular cross sections from PA pressures generated by variable tissue at the speed of sound (SoS). However, the use of ideal SoS constancy in general reconstruction is assumed to result in degradation of image quality. Sun et al. proposed to improve IVPAT image quality in tissues with variable SoS by simultaneously recovering SoS, AOED, and OAC from a sequence of measured time-varying pressures.^129^ The joint recovery is achieved by alternating forward simulations to minimize the error between measured and theoretical pressures. The results show that this method significantly reduces the normalized mean square absolute distance of the reconstruction.

Heart beating artifacts greatly impact cardiac reconstruction, necessitating algorithms to avoid such disturbances. For example, Taruttis et al. applied a clustering algorithm (k-means) to automatically separate single-pulse image sequences of multiple excitation wavelengths into clusters corresponding to different cardiac cycle phases.^130^ Each cluster was then subjected to spectral unmixing to obtain images of tissue-intrinsic chromophores at different cardiac phases, showing reduced sensitivity to motion compared to signal averaging without clustering. The results suggest that the correction method can be generally applied to multispectral optoacoustic tomography applications that are susceptible to motion artifacts such as respiration and heartbeat. A similar clustering algorithm was used in IVPA to mitigate motion artifacts of catheter pullback and heartbeat. Sun et al. reconstructed continuous images based on time-reversed clustered signal frames to represent the initial pressure distribution in the vessel cross-section.^131^ The method was shown to be computationally more efficient in motion correction compared with the image-based gating. For more rapidly demanded volumetric PAT techniques, such as the fast chimpanzee heart rate (400–600 beats per minute) cardiac imaging, Li et al. proposed a Fourier analysis on four-dimensional data to co-register independent sequences of cardiac volumetric PA images.^132^ The fundamental frequencies and higher harmonics of the respiratory and cardiac cycles can be clearly distinguished, which facilitated efficient retrospective alignment without additional readings.

Recent PAI systems can have volumetric frame frequencies of more than 100 Hz, and further improvements in temporal resolution are possible with partial data acquisition. However, for accurate reconstruction using compressed sensing methods, the acquired data must be known *a priori*. To overcome the limitation, Ozbek et al. proposed a machine learning method based on principal component analysis for high-frame-rate volumetric cardiac imaging using only a few tomographic PA projections.^133^ The training phase allowed efficient compression of cardiac motion information, which was subsequently used as prioritized information to reconstruct images from sparse samples at higher frame rates. The results show that image quality remains unchanged despite a 64-fold reduction in the data stream. Machine learning methods can also be used to eliminate motion artifacts in the cardiac cycle. For example, Sun et al. proposed a deep learning-based method for directly correcting motion artifacts in non-gated IVPA pullback sequences.^134^ The raw signal frames were classified into dynamic and static frames by clustering. Then, a neural network called motion artifact correction network was designed to correct the motion in dynamic frames. Although they trained and tested the network using a computer-generated coronary dynamic motion dataset, the results showed that the network could still directly correct motion in consecutive frames while preserving the original structure without discarding any frames. Improvements in longitudinal view visualization are demonstrated based on a quantitative assessment of inter-frame similarity.

Other method such as differential photoacoustic radar (DPAR) modes in the high-frequency domain was utilized by Choi et al., was successfully used for accurate molecular-specific assessment of vulnerable plaques.^135^ However, it is sensitive and specific only for spectroscopically defined imaging target cholesterol.

## Fluorescence imaging in cardiovascular diseases

Except for OCT and PAI, FLI also sees important applications in cardiovascular research, providing unparalleled insights into the molecular and cellular processes of the heart under both normal and pathological conditions. This imaging modality employs fluorescent markers that emit light upon excitation, enabling the visualization of dynamic biological processes in real time. The applications are extensive, ranging from mapping molecular interactions to assessing tissue viability and function during heart disease. High sensitivity and specificity of FLI make it indispensable for understanding cardiac pathophysiology and developing therapeutic strategies. Significant advances have been made in FLI techniques, including the development of novel fluorescent probes and sophisticated imaging systems tailored for cardiac applications. These innovations enhance the resolution and depth of cardiac imaging, facilitating detailed anatomical and functional analysis at both cellular and subcellular levels. This progress is crucial for advancing our understanding of cardiac biology and improving clinical outcomes in CVD management.^136^ In this section, we will summarize the applications of fluorescence imaging across various types of CVDs.

### FLI in coronary artery disease

As mentioned, CAD is the most common type of heart disease and involves the narrowing or blockage of the coronary arteries, usually due to atherosclerosis. FLI could be a valuable tool in studying CAD. For instance, Stein-Merlob et al. successfully achieved *in vivo* detection of designed nanoparticle deposition in atheroma by NIR FLI, as shown in Figure 9A.^5^^,^^137^ They investigated the relationship between impaired endothelial barrier function and atherothrombosis using nanoparticle-enhanced molecular imaging. Atherosclerosis was induced in rabbits through aortic balloon injury and a high-cholesterol diet. The rabbits were administered ultrasmall superparamagnetic iron oxide nanoparticles tagged with a NIR fluorophore, which accumulated significantly in atheroma. This accumulation was associated with enhanced endothelial permeability and was more pronounced in plaques that subsequently developed thrombosis following a pharmacological trigger.

**Figure 10:**
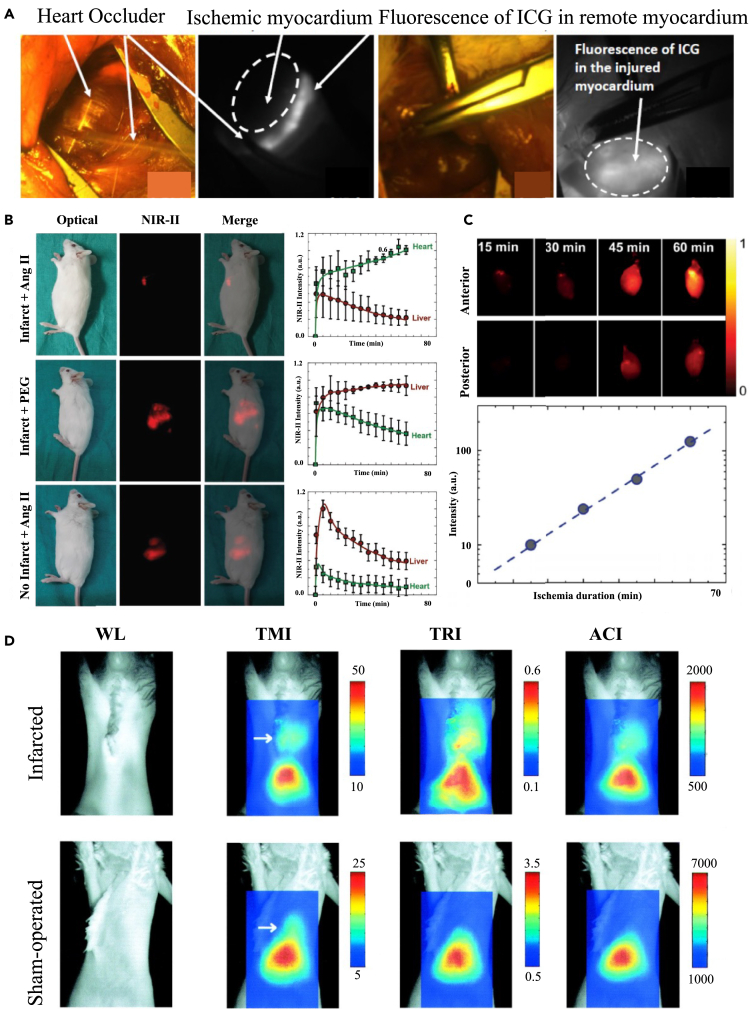
Nanoparticles-guided FL imaging of heart (A) Intravital visualization of the ischemic-reperfused area of the heart with ICG. From left to right, perfusion defects immediately after ICG administration (first two figures) and intravital epicardial ICG fluorescence. Reproduced with permission from.^140^ Copyright 2017 Optical Society of America. (B) Optical, NIR-II fluorescence, and merged images 10 min post-injection of Ag_2_S nanodots, showcasing three scenarios: top row - acute infarct mouse injected with AngII-functionalized Ag_2_S; middle row-acute infarct mouse injected with PEGylated Ag_2_S; bottom row-sham-operated mouse injected with AngII-functionalized Ag_2_S. The line charts show NIR-II luminescence intensity at the heart and liver of mice in three different situations. Reproduced with permission from.^141^ Copyright 2020 WILEY-VCH GmbH. (C) NIR-II fluorescence images of murine hearts under varying ischemia durations using Ag_2_S-ATII nanodots and NIR-II luminescence intensity from Ag_2_S-ATII nanodots in infarcted hearts plotted against ischemia duration. Reproduced with permission from.^142^ Copyright 2019 Springer Nature. (D) Planar *in vivo* fluorescence images overlaid on white light images show differences between an infarcted mouse and a sham-operated mouse (WL: white light, TMI: transillumination intrinsic image, TRI: transillumination ratio image, ACI: attenuation-corrected image). Reproduced with permission from.^143^ Copyright 2007 Wolters Kluwer Health.

Many studies thus far have been based on cellular aspects due to the depth limitation of FLI. The application of fluorescent molecular probes and biomarkers can achieve molecular-level diagnosis of CVDs through optical imaging. For example, Wang et al. emphasized the role of inflammation and oxidative stress in atherosclerosis, pointing to an increased demand for glutathione (GSH) to combat the excess reactive oxygen species (ROS). Two potential biomarkers, the GSH-hydrolyzed protein GGT and the ROS-derived hypobromous acid (HBrO), are identified as critical in predicting atherogenesis. To mitigate the risks of false-positive diagnoses from relying on a single biomarker, they introduced a novel fluorescent probe, C-HBrO-GGT, engineered to activate sequentially through GGT and HBrO actions, ensuring fluorescence only after specific conditions are met. The GGT + HBrO is the more sensitive biomarkers (about two times) of early atherosclerosis than conventional CD40. As shown in Figures 9B–10E, this sequence activation allows for precise detection of atherosclerotic plaques at early stages with FLI technology, even before they are visually detectable, providing an advanced tool for early warning and accurate localization of potential atherosclerotic developments.^138^

Jaffer et al. realized the utilization of magneto fluorescent nanoparticles (MFNPs), specifically dextran-coated NIR fluorescent ones, to image cellular inflammation in atherosclerosis using FLI techniques. On FL microscopy, MFNPs were found in cellular-rich areas of atheroma and colocalized with immunofluorescent macrophages over endothelial cells and smooth muscle cells. These results suggest that FLI with MFNPs can effectively monitor and quantify cellular distribution and inflammation in atherosclerosis, offering a promising tool for both research and potential clinical applications in assessing and managing this disease.^139^

### FLI in myocardial infarction

Myocardial infarction, also named heart attack, is often a result of severe CAD. FLI plays a significant role in understanding and managing heart attacks due to its ability to provide detailed visualizations of biological processes in real time. For instance, Sonin et al. visualized MI *in vivo* using ICG, a fluorophore.^140^ ICG accumulates around I/R injury areas due to increased vascular permeability, allowing the dye to extravasate, as shown in Figure 10A. ICG was intravenously infused during the late phase of ischemia. NIR fluorescence video captured the epicardial fluorescence of ICG, which matched the injured heart areas. Their study also founded that the ICG fluorescence method can measure the infarct size as accurate as the *ex vivo* traditional triphenyl tetrazolium chloride staining, suggesting that ICG fluorescence could serve as an alternative method for assessing MI both *in vivo* and *ex vivo*.

**Figure 11:**
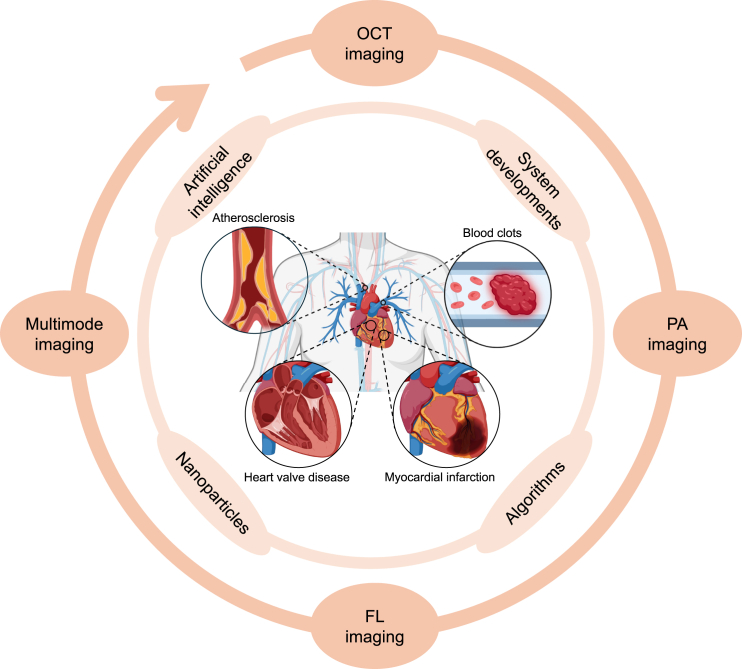
Graphic of the overall developments on optical imaging in CVDs

Nakayama et al. developed a surgical imaging system utilizing NIR fluorescent light, which has low autofluorescence, deep tissue penetration, low tissue scatter, and is invisible. By isolating visible and NIR light paths, this system allows simultaneous visualization of heart anatomy and/or function. In rat models, NIR fluorophores IR-786 and IRDye78 can be intravenously injected to provide real-time assessment of myocardial blood flow or perfusion during surgery.^144^

Mateos et al. designed a bio-functionalized NIR-emitting nanoparticles and employed them for *in vivo* imaging of the heart post-myocardial infarct.^141^ This approach utilized the fast acquisition capabilities of NIR-II FLI combined with the effective selective targeting of the nanoparticles, allowing for the rapid visualization of the infarcted heart within minutes of the infarction event, as shown in Figure 10B. The first figure in Figure 10B presents NIR-II fluorescence images illustrating the effective *in vivo* targeting of functionalized Ag_2_S-AngII nanoparticles. Compared with traditional methods (biochemical blood analysis ‘detection of cardiac enzymes’ or by ultrasound-assisted imaging) in diagnosing a heart attack at early times, the NIR-II fluorescence imaging based on functional nanoparticles can overcomes the disadvantages of time-consuming procedures or low spatial resolution. This development promises a cost-effective, swift, and precise method for *in vivo* imaging of the ischemic myocardium following an acute infarct.

The Ag_2_S nanodots have already been established as promising NIR emitting nanoprobes with low toxicity, high penetration, and high resolution for *in vivo* imaging. In addition to the research did by Fernández’s group,^141^ Ortgies et al.^142^ have investigated the application of Ag_2_S nanodots in accurately imaging damaged myocardium tissues post-MI induced by partial or global ischemia. As shown in Figure 10C, NIR-II images generated by ATII-functionalized Ag_2_S nanodots provide valuable information on the location and extent of myocardial tissue damage, facilitating the identification of occluded arteries and indirect evaluation of damage severity. Additionally, proof of concept whole-body imaging experiments further supports the potential future application of Ag_2_S nanodots for *in vivo* MI imaging.

Sosnovik et al. investigated noninvasive FLI of the heart using advanced transillumination and tomographic techniques to obtain *in vivo* images that can be synchronized with CMR.^143^ This research focuses on the uptake of CLIO-Cy5.5, a magneto fluorescent nanoparticle, by macrophages in myocardium affected by infarction in mice. After administering the particles post-surgery, imaging results showed enhanced contrast and fluorescence (about four times greater) in infarcted regions compared to control (shown in Figure 10D), confirming macrophage activity. These findings demonstrate the feasibility of noninvasively imaging myocardial macrophage infiltration and suggest potential for broader applications of FLI in cardiac research and clinical settings.

### FLI in heart valve disease

Valvular heart disease encompasses conditions that damage or cause defects in any of the four main heart valves: the mitral, aortic, tricuspid, or pulmonary valves. Specifically, the calcific aortic valve is a significant concern in this group. In calcific aortic valve disease (CAVD), calcium deposits accumulate on the aortic valve, a critical component that manages the flow of blood from the left ventricle to the aorta, the body’s main artery. This accumulation leads to the thickening and hardening of the valve, disrupting its ability to function properly. As a result, valve dysfunction such as stenosis or regurgitation occurs, contributing to the broader category of HVD. In addressing the diagnostic challenges posed by CAVD, FLI emerges as a promising technique. This method utilizes fluorescent probes that bind to the calcific deposits, allowing for precise visualization of the extent and pattern of calcification. This enhances the assessment and management of the disease, providing critical information that can guide therapeutic interventions. Baugh et al. designed a two-photon excited fluorescence (TPEF) imaging to analyze excised human CAVD valves and rat bones as well as calcific nodules in engineered gels.^145^ The authors also image and analyze the samples both before and after demineralization. This analysis led to the identification of an endogenous fluorophore associated with mineral levels. Building on this discovery, the authors developed a radiometric imaging technique that quantitatively indicates mineral presence in early-stage calcifications. This approach enables TPEF to perform non-destructive, high-resolution imaging of 3D tissue samples, crucial for detecting calcification in early and advanced disease stages.

Tandon et al. developed a mouse model for early detection of CAVD using a diet-based approach without genetic modification.^146^ Utilizing TPEF microscopy, they provided noninvasive and quantitative insights into various pathophysiological stages like calcium deposition and collagen remodeling. The mice were monitored through various imaging and molecular techniques, demonstrating that those on a pro-calcific diet showed early signs of lipid deposition and calcification. Lower TPEF autofluorescence ratios at specific valve locations correlated with advanced pathological features, suggesting TPEF metrics as a valuable tool for early detection and monitoring of CAVD progression.

## Other optical imaging modalities

As conventional optical imaging modalities face the limitation of low penetration depth for *in vivo* applications, novel optical imaging techniques have been developed. These include afterglow imaging, ultrasound luminescence, and X-ray excitation luminescence. Afterglow imaging offers prolonged light emission after the excitation source is removed, enabling extended imaging times and reducing background noise, which has been applied in various diseases, such as tumor imaging,^147^^,^^148^ brain disorder imaging,^149^ and drug delivery and tracking.^150^ Ultrasound luminescence allows for deep tissue imaging by using ultrasound waves, which penetrate deeper than visible light, thus providing high-resolution images without the scattering issues typical of conventional optical methods. The ultrasound triggered luminescence in biomedical applications remains in its early stages of development.^151^^,^^152^ X-ray excitation luminescence combines the high penetration depth of X-rays with sensitive luminescent signal detection, allowing for detailed visualization of structures within thick samples that are challenging to image with traditional optical techniques. The X-ray excitation luminescence has also been used in a variety of biomedical applications, such as lymph node imaging,^153^ *in vivo* bioimaging,^154^ and monitoring of thrombosis.^155^

However, so far, these imaging modalities applied in cardiovascular disease are scarcely explored. A photochemical afterglow implant with strong intensity and long lifetime was developed for embolization and imaging. Injected into the abdominal aorta of mice, it forms a hydrogel to block the vessel, allowing visualization of the embolism and monitoring of the embolization effect, reducing autofluorescence interference.^156^ Zhang et al. designed inexpensive organic-doped long-wavelength room temperature phosphorescent materials with benzo[c][1,2,5] thiadiazole. These materials, with good tissue penetration (10 mm), successfully imaged atherosclerotic plaques with a signal-to-back- ground ratio of 44.52 based on phosphorescence emission, offering a new method for imaging cardiovascular diseases.^157^

## Multimode imaging of cardiovascular diseases

As mentioned earlier, OCT has high resolution, PAI has desirable balance between resolution and imaging depth, and FLI is able to specifically target certain molecules, but each of them has its shortages. Therefore, multimodal cardiac imaging can provide diagnostic information from various aspects and hence is desired to provide more comprehensive and accurate vascular assessments.

For example, the fusion of OCT and FLI is expected to provide simultaneous imaging of vascular morphology and molecular functions. The Fluorescence and OCT signals can be acquired using a single optical fiber and lens, offering more comprehensive information for the diagnosis and risk assessment of CVDs.^158^ In 2021, Zanchin et al. reported a compelling case demonstrating the good concordance between NIRS, OCT, and postmortem histopathology for the detection of lipid-rich plaques in coronary arteries.^159^ The researchers performed multimodality imaging using NIRS-IVUS and OCT in the LAD and right coronary artery (RCA) of a patient with acute ST-elevation myocardial infarction (STEMI).^160^ The NIRS chemo gram revealed lipid-rich plaques in both the LAD and RCA, which was confirmed by matched OCT and IVUS images. Unfortunately, the patient died on Day 5 due to ventricular myocardial rupture and cardiac tamponade. Postmortem histological examination of these vessels further validated the assessment made by *in vivo* intracoronary imaging. This unique cardiovascular case strongly confirms the consistence of information between NIRS, OCT, and postmortem histopathology for lipid detection.

Several studies have also demonstrated the potential of combining OCT and PAI for the assessment of atherosclerotic plaques. Shang et al. revealed the optical absorption and scattering properties of vascular plaques by combining PAT and OCT, which in turn was used to distinguish the composition and structure of plaques.^161^ PAT obtained lipid information through optical absorption differences, while OCT obtained collagen imaging through scattering differences. The combined PAT and OCT technique was demonstrated to be a potentially viable method for detecting the composition and structure of the lipid core and fibrous cap in atherosclerosis. In 2018, Mathews et al. developed an intravascular probe with both OCT and PAI capabilities.^162^ The probe integrates a commercial OCT catheter with a highly sensitive fiber-optic ultrasound sensor. OCT imaging provides fine structural information of the vessel wall, while PAI has selective absorption for tissue components, enabling molecular imaging contrast. The results showed that the dual-modality probe had good imaging performance for absorbing dyes and stents, with a lateral resolution of 18–40 μm and an axial resolution of about 45 μm. This work is the first to prove that a commercial OCT catheter can also be used to transmit PA excitation light, paving the way for new approaches in IVPA imaging. To further upgrade, Leng et al. reported a novel multi-spectral intravascular tri-modality imaging system that integrates PA, US, and OCT imaging modalities in 2021.^163^ The system employs a 0.9 mm miniature catheter to achieve 360-degree continuous rotational scanning, simultaneously acquiring both macroscopic and microscopic structural information of the vessel wall, as well as compositional information. Furthermore, the multi-wavelength PA mode enables the identification and quantification of lipids. This is the first reported tri-modality imaging system capable of performing 360-degree rotational scanning within blood vessels, providing a new imaging platform for the accurate diagnosis of high-risk plaques. In the context of intravascular imaging, IVPAI provides high-resolution and high-penetration images of intramural hematomas (IMH) of varying depths, making it particularly suitable for imaging deep clots associated with imaging artifacts, while IVOCT can easily visualize the dual-lumen morphology of the vessel wall and thus identify intimal tears. Then, the combination of IVPAI with IVOCT inspires an intravascular dual-mode endoscopic system, which utilizes a probe with an outer diameter of 1.0 mm, for identifying spontaneous coronary artery dissection (SCAD) disease models.^164^ In this dual-mode endoscopic system, it provides excellent information on vascular anatomy and enables detection of deep IMH. This is the first time that OCT and PA have been combined for the detection of SCAD. In 2023, Zhou et al. proposed a conceptually novel method for treating atherosclerotic plaques using OAI and OCT-guided lipid-selective ablation.^165^ The paper investigated the mechanisms of pulsed laser-tissue interaction and established an ablation model to guide the optoacoustic ablation process. OCT was used to observe changes in fibrous caps and vessel walls before and after ablation, with histology used for validation. The paper demonstrated its ability to remove plaques precisely and effectively through experiments on plaque-mimicking phantoms and aortic samples from ApoE-KO mice. This is the first report on using OAI and OCT for theragnostic research on atherosclerotic plaques.

The fact that PAM-specific imaging of blood vessels can be used to visualize blood vessels and monitor their changes over time also allows for more combinations. For example, the combination of OCT with PAM enables dual-mode functional imaging in heart.^166^ The PA mode employs a kinetic energy optical sensor with a large imaging window. This imaging window enables direct reflection mode operation and seamless integration of OCT as a second imaging mode. Functional extensions to the OCT-PAM system include Doppler OCT and spectral PAM (sPAM). This functional noninvasive imaging system was applied to zebrafish larval imaging, demonstrating its ability to extract morphological and hemodynamic parameters in small animals that are critical for physiology, pathophysiology and drug response studies in preclinical imaging. Ahn et al. proposed a novel fully integrated PAM and photoplethysmography (PAM-PPG) system for visualizing hemodynamic features.^167^ The system can simultaneously acquire vascular images of human fingers (via PAM) and blood volume changes (via PPG). The heart rate is then determined from changes in the PA signal, which closely matches the PPG signal. PAM-PPG may become a useful clinical tool in several clinical areas such as cardiology and endocrinology.

Last but not the least, the fusion of PAI and FLI also enables new functionality. For example, excessive blood delivery into the plaque causes intraplaque hemorrhage (IPH), which influences the formation and retention of cholesterol crystals, hemoglobin and oxidative enzymes as well as protein hydrolysis activity in the blood. IPH is also one of the key determinants of plaque vulnerability. Zhan et al. proposed a 1-mm-diameter dual-mode endoscopic probe for simultaneous detection of lipid cores and IPH within plaques, which consisted of PAI and near-infrared autofluorescence imaging (NIRAFI).^168^ The authors used *in vitro* phantom and *in vivo* rabbit aortic experiments to demonstrate the feasibility and capability of the dual-mode probe for imaging IPH and lipid core expansion. The results suggest that dual-mode endoscopy has the potential to accurately detect IPH in atherosclerotic plaques. Most recently, Chen et al. reported a phosphatidylserine-specific peptide, CLIKKPF-functionalized carbon dot nano-enzymes (pep-CDs), which can be used for specific and highly efficient non-invasive treatment of atherosclerosis and enabled multimodal PAI and FLI imaging visualization.^169^ The pep-CDs not only inherit the intrinsic properties of carbon dots (CDs), including deep red fluorescence emission, PA response, superoxide dismutase-like antioxidant and anti-inflammatory activities, but also have the ability to target recognition on foam cells and localize them on plaques due to the specific interactions of CLIKKPF with phosphatidylserine on the outer surface of foam cell membranes. The targeted localization effect of pep-CDs greatly facilitates the effective aggregation of CDs in plaques, thus maximizing the therapeutic effect of CDs in atherosclerosis. Furthermore, it can be used to image plaque morphology and monitor in real time the progression of atherosclerotic pathology due to differences in foam cell content. This work highlights the advantages of active target-assisted probes and multimodal imaging in atherosclerosis therapeutic applications.

## Conclusion and perspectives

In this review, we have summarized recent advancements of optical imaging, especially including OCT, PAI, FLI, afterglow luminescence and their multimodal approaches, for the diagnosis application of CVDs. OCT has demonstrated remarkable capabilities in providing high-resolution cross-sectional imaging of vascular morphology and tissue histology, enabling the identification of vulnerable plaques, myocardial injury, heart valves dysfunction and guiding interventions; PAI has shown promise in assessing myocardial oxygenation, blood perfusion, and molecular signatures, offering non-invasive characterization of ischemic heart disease; various versions of FLI have facilitated the visualization of specific molecular targets involved in cardiac pathologies, such as inflammation, fibrosis, and apoptosis, aiding in early detection and targeted therapy. However, the limitations of a single imaging modality restrict the diagnostic specificity and accuracy of CVD. The integration of multiple imaging modalities, such as combining OCT with PAI or FLI, has provided complementary information and parameters from multiple aspects ranging from molecular metabolism to macroscopic structure, enhancing the accuracy and comprehensiveness of CVD diagnosis and treatment. We have compiled and listed some relevant studies in recent years, which we hope to provide a reliable basis and vision for heart and cardiovascular research (Table 2).

**Table 2:** Optical imaging modalities in cardiovascular diagnosis and treatment

Imaging Modality	Systems	Diseases	Application Scenarios	Novelty and findings	Algorithms
OCT	• Polarization-sensitive OCT (PS-OCT)^51^^,^^52^ • Intravascular OCT (IVOCT)^57^ • OCT angiography (OCTA)^62^	CAD	Identifying histological features of atherosclerotic plaques	• Plaque morphology, composition, and vulnerability visualization, both *ex vivo*^23^^,^^24^^,^^25^^,^^26^ and *in vivo*^27^^,^^28^	• CNNs improve efficiency and accuracy of diagnosis^57^^,^^58^^,^^59^^,^^60^^,^^61^^,^^62^ • Data enhancement using cGANs can significantly improve the performance of deep learning models when dealing with limited datasets^63^ • Transformer architectures show superior performance in segmentation and features extraction^64^^,^^65^^,^^66^^,^^67^
			Identifying of hemodynamically significant stenoses	• Higher diagnostic accuracy for stenosis^30^^,^^31^	
			Evaluating the efficacy of therapies	• Proprotein convertase subtilisin/kexin type 9 (PCSK9) promoting plaque stabilization^29^	
			Guiding PCI and immediate evaluation	• Facilitating stent selection, positioning, and deployment^33^^,^^34^^,^^35^ • immediate evaluation after the stent implantation^36^^,^^37^^,^^38^	
			Evaluating follow-up after coronary stent implantation	• Assessing stent apposition and healing^39^^,^^40^^,^^41^^,^^42^	
		MI	Analyzing myocardial histology and function	• Quantitative analysis of important structural features such as myocardial fiber orientation, density, and collagen fiber distribution^43^^,^^44^ • Differentiating adipose tissue, fibrosis^47^^,^^49^ and micro vessels^48^ features.	
			Guiding ablation therapy for atrial fibrillation	• Human pulmonary vein-atrial junction imaging and guiding^43^ • Real-time monitoring of lesion formation, predicting steam pops, and reflecting the contact state and angle between the catheter and tissue^46^	
		HVD	Real-time imaging of heart valves with high resolution	• The aortic and mitral valves imaging in a mouse model^50^ • EndMT process dynamic imaging in the outflow tract cushions of chicken embryonic hearts^51^	
			Guiding mitral valve dysfunction diagnosis and surgical treatment	• Studying birefringence properties of human mitral valve CTs^52^ • Monitoring in mital value surgery^53^	
		CHD	Evaluating CHD morphology and function	• Assessing pulmonary artery wall changes in human^56^ • Evaluating the cardiac activity and gene regulation of insect embryos^54^^,^^55^	
PAI	• Optical resolution PAM (OR-PAM)^72^^,^^73^ • Linear-array PAT^74^^,^^75^ • Hemispherical-array PACT^76^^,^^77^^,^^82^ • Ring-array PACT^78^ • Intravascular PAE (IVPAE)^83^^,^^84^^,^^85^^,^^86^ • IVPAE + ablation, including laser,^87^^,^^90^ radiofrequency,^88^ and ultrasound^89^ ablation • Robotic PA system^93^^,^^94^ • Flexible optoacoustic blood "stethoscope" (OBS)^97^ • Multispectral photoacoustic imaging (MSPAI)^100^	CAD	Characterizing lipid-rich plaques spatially and quantitatively	• Assessment of pixel-based LRC in the vessel wall^98^ • Evaluating the efficacy of medical therapies through lipid plaque monitoring^102^	• DAS-based adaptive beamforming algorithm effectively reduces time-varying noise and increases the specificity of myocardial in PA^124^^,^^125^^,^^126^^,^^127^ • Physiological signal-gated PAI technique with motion compensation applied to estimate subtle oxygen saturation in ischemic vessels^128^ • Reconstruction with variable SoS, that recovering from measured time-varying pressure sequences, greatly reduces the normalized mean-square absolute distance^129^ • Clustering^130^^,^^131^ Fourier analysis-based^132^ algorithms and deep learning methods^133^^,^^134^ are successfully used to mitigate motion artifacts of catheter pullback and heartbeat. • Differential photoacoustic radar modes in the high-frequency domain are effective in assessing cholesterol of vulnerable plaques^135^
			Real-time monitoring of plaque morphology and components	• Detecting the presence of hemorrhage in surgery^99^ • Distinguishing new from old hemorrhages and cholesterol features using unmixed technique^100^	
			Targeting probes for enhanced PAI of atherosclerotic plaques	• Nanoprobe OPN Ab/Ti_3_C_2_/ICG^114^ • Ratiometric semiconducting polymer nanoparticle (RSPN)^101^ • DCP liposomes (DOPS and Cypate-PC)^106^ • lipid-unlocked CTB response probe (L-CRP)^107^ • HDS-GGT^108^	
			Targeting probes for treatment of CADs	• Therapeutic nanoprobes using hydrogen peroxide (H_2_O_2_) activation mechanisms,^109^ multichannel regulation of lipid dissolution and metabolism,^110^^,^^111^ and immune microenvironment rebuilding strategy,^112^ etc.	
		MI	Timely and accurate diagnosis and longitudinal monitoring	• Showing gradual enlargement of the infarcted area with necrosis and fibrosis by longitudinal observation^114^	
			Accurate delineation of MI boundaries	• Calculating the "the ratio of the areas of the power spectral densities (R_APSD_) " of different regions using dual wavelength photoacoustic spectral analysis^114^	
			Targeting probes for enhanced PAI to detect infarcted areas and help diagnose MI	• Fibronectin-targeted nanoparticles (CREKA-ICG-LIP NPs)^115^ • Ultrasmall single-stranded DNA (ssDNA)/metal ion complexes nanoprobe^116^ • Collagen-targeted nanoplatform CNA35-GP@NPs.^117^	
			Targeting probes for enhancing therapeutic efficacy against I/R injury	• Gold-selenium core-shell nanostructures AS-I/S NCs^118^ • PEGylated allomelanin nanoparticles AMNPs@PEG^128^	
		Diabetic cardiomyopathy	Study of vascular microcirculatory dysfunction	• Skeletal muscle microvascular dysfunction precedes significant changes in the heart, such as thinning, fibrosis, or hypertrophy, in both male and female patients^120^	
		Acute hyperglycemia	Monitoring microvascular changes to avoid cardiovascular risk	• Acute hyperglycemia causes transient vasoconstriction of the arterial vasculature^121^	
		Arrhythmias	Acquiring the propagation of electromechanical waves in heart	• Sparse optoacoustic sensing using cardiac volumetric motion enables ultra-fast four-dimensional imaging of cardiac mechanical wave propagation throughout the beating mouse heart^122^	
		Cardiac arrest	Quantifying neurovascular oxygenation and cardiac function after cardiac arrest and resuscitation	• Quantification of biomarkers such as neurovascular coupling, overall cardiac longitudinal strain, and ejection fraction can identify correlations between neural and cardiovascular parameters, which may help predict cardiac function from an imaging perspective^123^	
FLI	• NIR FLI^5^^,^^137^^,^^139^ • NIR-II FLI^141^^,^^142^ • Two-photon excited fluorescence (TPEF) microscopy^145^^,^^146^	CAD	Investigating the relationship between impaired endothelial barrier function and atherothrombosis	• The ultrasmall superparamagnetic iron oxide nanoparticles accumulated significantly in atheroma^5^^,^^137^	N/A
			Predicting atherogenesis with inflammation and oxidative stress biomarkers	• C-HBrO-GGT allows to activate sequentially through GGT and HBrO actions, ensuring early diagnosis of atherosclerosis than CD40^138^	
			Monitoring and quantifying cellular distribution and inflammation in atherosclerosis	• Dextran-coated magneto fluorescent nanoparticles show accumulation in endothelial cells and smooth muscle cells rich areas of atheroma^139^	
		MI	Visualizing injured heart areas with enhanced dyes	• ICG^140^ • Ag_2_S-AngII NPs^141^ • Ag_2_S nanodots^142^ • CLIO-Cy5.5.^143^	
			Assessing myocardial blood flow or perfusion during surgery	• IR-786 and IRDye78^144^	
		HVD	Diagnosing calcific aortic valve disease (CAVD)	• Allowing for precise visualization of the extent and pattern of calcification and guiding therapeutic interventions^145^ • Quantitative analyzing of various pathophysiological stages like calcium deposition and collagen remodeling^146^	
X-ray-excited luminescence (XEL)	/	Thrombotic carotid	Diagnosing thrombosis	• Achieving high-efficiency XEL imaging of the early thrombosis on the basis of *in situ* elevated thrombin levels with the use of core-shell lanthanide-dopedscintillator nanocrystals^155^	N/A
Afterglow luminescence	/	Abdominal aorta blocking	Visualizing the embolism and monitoring of the embolization effect	• A photochemical afterglow implant with strong intensity and long lifetime was developed for embolization and imaging^156^	N/A
		CAD	Imaging atherosclerotic plaques	• Inexpensive organic-doped long-wavelength room temperature phosphorescent materials with benzo[c][1,2,5] thiadiazole was designed for visualizing atherosclerotic plaques with high signal-to-back-ground ratio.^157^	
Multimodal imaging	OCT + FLI	CAD	Diagnosis and risk assessment of CVDs	• Imaging of vascular morphology and molecular functions and detecting of lipid-rich plaques in coronary arteries^158^^,^^159^	N/A
	OCT + FLI + IVUS	MI	Imaging of a patient with acute STEMI	• Lipid-rich plaques in both the LAD and RCA were visualized clearly^160^	
	OCT + PAT	CAD	Distinguish the composition and structure of plaques	• Detecting the composition and structure of the lipid core and fibrous cap in atherosclerosis^161^	
	IV OCT + PA	CAD	Detecting vessel wall structure and tissue components	• The first commercial OCT catheter with transmitted PA excitation light^162^	
	IV OCT + PA + US	CAD	Identifying and quantification of lipids	• The first reported 0.9 mm miniature catheter tri-modality imaging system capable of performing 360-degree rotational scanning within blood vessels^163^	
	IV OCT + PA	SCAD	Detection of deep IMH	• The first OCT and PA combined detection of SCAD.^164^	
	IV OCT + PA	CAD	Guiding lipid-selective ablation	• The first using OAI and OCT for theragnostic research on atherosclerotic plaques^165^	
	OCT + PAM	Healthy heart	Imaging of zebrafish larval	• Doppler OCT and spectral PAM dual-model system shows its ability extract morphological and hemodynamic parameters in small animals^166^	
	PAM + PPG	Healthy heart	Visualizing hemodynamic features	• Simultaneously acquiring vascular images of human fingers (via PAM) and blood volume changes (via PPG), and calculating the heart rate^167^	
	IV PA + NIRAFI	CAD	Simultaneous detection of lipid cores and IPH within plaques	• The dual-mode endoscopy has the potential to accurately detect IPH in atherosclerotic plaques^168^	
	PAI + FLI	CAD	Non-invasive visualization and treatment of atherosclerosis	• The pep-CDs has the ability to target recognition on foam cells and localize them on plaques, achieving simultaneous imaging and treatment of atherosclerosis^169^	

It also should be noted that most studies thus far are based on human samples or small animal models and are at the stage of basic biomedical research. Further validation studies and large-scale clinical trials are needed to determine the clinical efficacy, accuracy, and cost-effectiveness of optical imaging technologies. Thus, in future, the *in vivo* visualization of CVDs will draw inspiration and direction from the development of increasingly sophisticated functional imaging systems, safer and more stable optical imaging agents, hotspot AI integration, wearable electronics, and so forth. For example, volumetric PAI, while allowing for noninvasive acquisition of cardiac activity in live small animals, is still not available for large animals or mammals at cardiac depth. Extending the imaging depth and field of view of those imaging implementations remains a critical challenge to date. Great efforts are needed to enhance deep-tissue penetration to overcome light scattering influence. The development of fiber-optic imaging^170^^,^^171^^,^^172^ and optical wavefront shaping techniques^173^ may empower future endoscopic or inserted optical microscopy for live vascular and biopsy imaging on the cardiac surface minimal-invasively. Exogenous probes have been subject to safety adjustments in the clinic. Therefore, the exploration of more biologically safe molecular probes targeting cardiomyocytes or macrophages, for example, is also a priority. Electrical signals are also a significant physiological signal. In recent years, optical imaging has gained popularity in the study of brain voltage imaging^70^^,^^174^ and neurovascular coupling^175^; however, it has yet to be explored in the field of electro cardiology. This may represent a major area of interest for future research. Convenient medical care that combines robotics and flexible electronics is also a much-needed direction and product for the next phase. Additionally, the standardization and optimization of quantitative analysis methods are crucial for accurate and reproducible measurements. Many algorithms are currently proposed that offer some enhancements for reconstructing images and quantification. However, there are no specific standards to normalize and realize these data. Developing more robust algorithms and protocols, such as novel deep learning model, for quantitative analysis and prediction will improve the clinical utility and accuracy of optical imaging in CVD diagnosis.

Bridging the gap between research and clinical implementations, relevant policies need to evolve in tandem. To facilitate the adoption of those imaging technologies in CVD management, healthcare providers and policymakers should also play a role in the progress. There are several possible strategies to facilitate the technology applications, such as establishing clear and streamlined regulatory pathways for new imaging technologies, developing and implementing reimbursement policies that cover the costs associated with these advanced imaging technologies that will encourage healthcare providers to adopt these technologies without financial barriers, creating standardized protocols and clinical guidelines for the use of OCT, PAI, and FLI in CVD management, providing funding and incentives for research and development of advanced imaging technologies, implementing training programs for healthcare professionals to ensure they are proficient in using these advanced imaging technologies, promoting the integration of these imaging technologies into routine clinical practice for cardiovascular disease diagnosis and management, lunching public awareness campaigns to educate patients about the benefits of advanced imaging technologies, encouraging collaborative research between academic institutions, healthcare providers, and technology developers.

## Acknowledgments

We acknowledge the supporting of the 10.13039/501100001809National Natural Science Foundation of China (81930048), Guangdong Science and Technology Commission (2019BT02X105), Hong Kong Innovation and Technology Commission (GHP/043/19SZ, GHP/044/19GD), Hong Kong Research Grant Council (15217721, R5029–19, C7074-21GF), and 10.13039/501100004377Hong Kong Polytechnic University (P0038180, P0039517, P0043485, 1-CEB1). Figure 11 is created in BioRender.com.

## Author contributions

W.P., C.Y., and Y.Z. prepared the figures for the manuscript and wrote the first version of the manuscript. L.N. and P.L. funded and supervised the project. All authors contributed to the revision and proofreading of the manuscript. All authors have read and agreed to the published version of the manuscript. W.P. and C.Y. contributed equally to this work.

## Declaration of interests

The authors report no competing interests.
